# Valproate regulates inositol synthesis by reducing expression of *myo*-inositol-3-phosphate synthase

**DOI:** 10.1038/s41598-023-41936-2

**Published:** 2023-09-08

**Authors:** Kendall C. Case, Rachel J. Beltman, Mary Kay H. Pflum, Miriam L. Greenberg

**Affiliations:** 1 Department of Biological Sciences, Wayne State University, Detroit, MI 48202 USA; 2 Department of Chemistry, Wayne State University, Detroit, MI 48202 USA

**Keywords:** Saccharomyces cerevisiae, PCR-based techniques, Immunoblotting, Immunoprecipitation, Blotting, Electrophoresis, Protein enrichment, Western blot, Mass spectrometry, Protein design, Kinases, Phosphoproteins, Phosphorylation, Molecular biology, Post-translational modifications

## Abstract

Inositol depletion is a hypothesized mechanism of action of mood stabilization drugs used in the treatment of bipolar disorder. It was previously reported that the mood stabilizer valproate (VPA) increased phosphorylation of *myo*-inositol-3-phosphate synthases (MIPS), the rate limiting enzyme of inositol synthesis. Phosphosites were identified and examination of site-directed mutants suggested that phosphorylation leads to decreased enzymatic activity. In this study, we examined the extent of MIPS phosphorylation in response to VPA and used two interaction screens to identify protein kinases that interact with MIPS. Using an epitope tagged MIPS construct, we determined the fraction of phosphorylated MIPS to be very low (less than 2% of total), and we could not detect phosphorylation of untagged MIPS in response to VPA. In vitro analyses of phosphorylation revealed that putative protein kinases, PKC and CKII, have low specificity toward MIPS. These findings suggest that VPA likely depletes inositol via a mechanism other than MIPS phosphorylation. Consistent with this, mRNA levels of the MIPS-encoding gene *INO1* and MIPS protein levels were significantly reduced during the mid-log growth phase in response to VPA treatment. These findings suggest that the mechanism whereby VPA causes inositol depletion is by reducing expression of the rate-limiting enzyme MIPS.

## Introduction

Integral to many regulatory mechanisms and signaling cascades, inositol and its derivatives are essential for cell viability. Most eukaryotic cells can take up inositol from the extracellular environment through transmembrane transport proteins or synthesize it de novo from glucose-6-phosphate. *Myo*-inositol (referred to as inositol in this article) is the most abundant inositol isoform. This six-carbon cyclitol sugar is the precursor to inositol-containing lipids and soluble inositol phosphates^1,2^, which play extensive roles in cellular regulatory mechanisms, including DNA transcriptional regulation^2–4^, metabolism^1,2,5,6^, organelle membrane labeling and trafficking^7,8^, signal transduction^1,6,8^, and others. Disequilibrium of inositol levels is associated with a significant number of diseases^1^, which is unsurprising considering its extensive role in regulatory pathways. One such disease is bipolar disorder, which is linked to over-excitability of certain neuronal networks hypothesized to be buffered by decreasing inositol-mediated signaling cascades ^9^.

Bipolar disorder is a psychiatric disorder characterized by manic and depressive episodes in which mood, energy, and motivation are drastically altered. It is highly prevalent worldwide (1–5%) and has the highest suicide rate of any psychiatric disorder^10^. Afflicted individuals also have a higher than normal incidence of obesity and related diseases, which contribute to poor health and related complications^1^. To help improve the quality of life and reduce mood swing frequencies, the condition is often pharmaceutically treated using mood stabilizers, the most common of which are lithium and valproate (VPA); however, other anticonvulsants and antipsychotic drugs have also been used to help stabilize mood swings^1^. Treatments can have adverse effects and are not universally effective. Like most psychiatric disorders, the therapeutic mechanisms of action that lead to mood stabilization are not understood, which constitutes a major barrier to the development of better treatments.

As the first approved treatment for bipolar disorder, lithium has been used for decades and is one of the most prescribed mood stabilizing drugs^11,12^. The finding that lithium causes inositol depletion led Michael Berridge and colleagues to propose the inositol depletion hypothesis, which suggests that mood stabilizers therapeutically reduce/buffer abnormally high IP_3_-induced Ca^2+^ release from the mitochondria of hyperexcitatory neurons (proposed as the underlying dysfunction in bipolar disorder patients)^1,9,13,14^. The first and rate-limiting step of inositol synthesis is the conversion of glucose-6-phosphate to inositol-3-phosphate by the catalytic action of *myo*-inositol-3-phosphate synthase (MIPS). The second step is dephosphorylation of inositol-3-phosphate by inositol monophosphatase (IMPase) to produce inositol. Lithium directly inhibits IMPase activity^15,16^ by displacing the Mg^2+^ cofactor(s)^17,18^, which consequently decreases inositol. Interestingly, VPA, originally used to treat epilepsy, also leads to inositol depletion^1,19–23^. However, VPA does not affect IMPase but indirectly inhibits MIPS^20,22,23^. Additionally, another anticonvulsant and mood stabilizer, carbamazepine, has also been shown to deplete inositol^1,24^, further lending support to the inositol depletion hypothesis, as three structurally distinct drugs target the same pathway to produce similar therapeutic effects. Furthermore, lithium and VPA both inhibit GSK3β, which is required for optimal MIPS activity in yeast^1,25–27^.

In yeast, MIPS is encoded by the gene *INO1* and its expression is heavily modified by the well-characterized Henry Regulatory Circuit in response to inositol^1^. However, this transcriptional regulatory circuit was previously thought not to be the mechanism of VPA-mediated inhibition of MIPS as levels of *INO1* increased following long-term treatment with VPA^19,20,23,26^. Rather, because site-directed mutagenesis of putative phosphorylation sites affected MIPS activity, it was suggested that MIPS was inhibited due to phosphorylation by an as yet unidentified protein kinase regulatory mechanism activated by VPA^19^. The current study set out to identify the protein kinase(s) responsible for MIPS phosphorylation and to determine the overall effects of MIPS phosphorylation on de novo inositol synthesis. Employing two screens to capture protein kinase interactions, we identified a small number of protein kinases that might be responsible for MIPS phosphorylation; however, no protein kinases were commonly identified by the two screens. We then examined in vivo phosphorylation of MIPS in protein kinase deletion mutants. Surprisingly, phosphorylation was only observed when MIPS was tagged with an Xpress epitope, described previously^19^. Mass spectrometry (MS) proteomic analysis of this purified protein identified phosphorylation of the tag itself, while endogenously expressed MIPS (which lacks the tag) had undetectable levels of phosphorylation. This suggests that phosphorylation in response to VPA is artificially elevated due to the presence of the tag. Overall, these findings indicate that inhibitory phosphorylation of MIPS is unlikely the mechanism of action of VPA-induced inositol depletion. Because *INO1* is highly regulated transcriptionally by the Henry Regulatory Circuit^1^, we examined the temporal response of MIPS protein and *INO1* mRNA expression to VPA. Our findings indicate that the mechanism whereby VPA reduces inositol synthesis is that of reduced *INO1* expression during the mid-log growth phase.

## Results

### 6xHis-Xpress-MIPS is rapidly phosphorylated in response to VPA treatment

**Figure 1:**
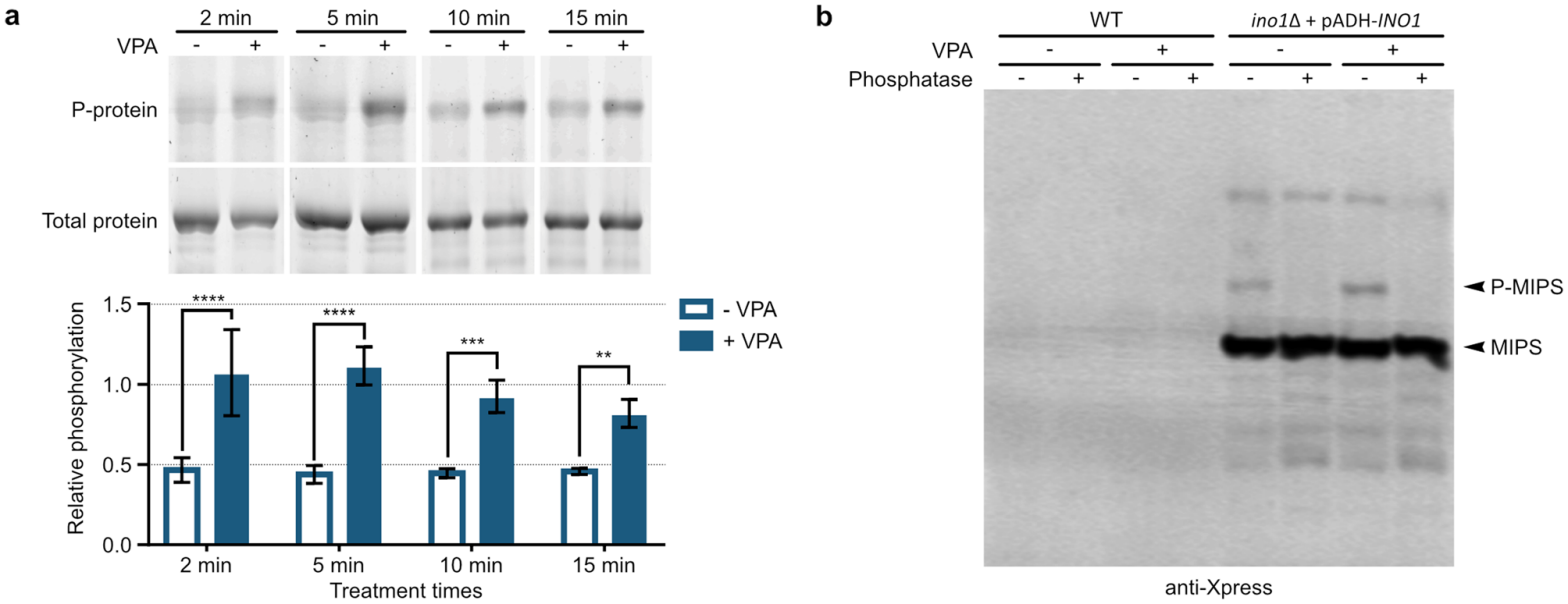
Temporal response (**a**) and fraction (**b**) of MIPS phosphorylation following VPA treatment. (**a**) 6xHis-Xpress-MIPS was purified from yeast treated with or without 1 mM VPA for 2, 5, 10, and 15 min (n = 3). Relative phosphorylation of purified proteins was evaluated by running on SDS-PAGE gels followed by tandem staining with Pro-Q™ Diamond phosphoprotein stain then SYPRO™ Ruby total protein stain (top; representative of three replicates, see uncropped gel images in Supplementary Fig. S1a online). Bands were quantified and phosphorylated MIPS was normalized to total MIPS (bottom; SD, two-way ANOVA; *p < 0.05, **p < 0.01, ***p < 0.001, ****p < 0.0001). (**b**) WT yeast expressing untagged MIPS or 6xHis-Xpress-MIPS were treated with or without 1 mM VPA for 5 min in the absence of inositol. Cells were lysed and WCEs were treated with or without phosphatase. Samples (10 µg total protein) were then analyzed by Zn^2+^ Phos-tag WB, where phosphorylated proteins (P-MIPS) migrate slower than unphosphorylated. WT expresses untagged MIPS and was used to verify the specificity of the antibody. The lower molecular weight bands below the MIPS band are likely degradation by-products. See uncropped/unedited membrane image in Supplementary Fig. S1b online.

Regulation of protein activity by phosphorylation is a labile and rapidly occurring process. Previous reports tested in vivo MIPS phosphorylation only after 1 h of VPA treatment. In order to determine the temporal response of VPA-induced MIPS phosphorylation, we employed a yeast strain constitutively expressing 6xHis-Xpress tagged MIPS (6xHis-Xpress-MIPS) from a low-copy number vector (pADH-*INO1*)^19^. Cultures were grown to mid-log growth phase and then treated with or without VPA for 2, 5, 10, and 15 min (2 min was the fastest time cells could be collected after addition of VPA). 6xHis-Xpress-MIPS was purified from whole cell extracts (WCEs) then analyzed by SDS-PAGE, which was stained in tandem with Pro-Q™ Diamond phosphoprotein and SYPRO™ Ruby total protein stains. There was an immediate increase in MIPS phosphorylation within 2 min of VPA treatment (Fig. 1a). Notably, the levels of phosphorylation appeared relatively low and did not increase at later timepoints but was otherwise maintained. While this methodology determined that the relative abundance of phosphorylation was elevated, we wished to determine the percent of phosphorylated protein, assuming that a significant fraction of MIPS would have to be phosphorylated to alter the rate of inositol synthesis. To do so, we utilized Zn^2+^ Phos-tag gel and Western blot (WB) analyses. Phos-tag gels reduce the mobility of phosphorylated proteins, causing an upward shift, separating them from their non-phosphorylated counterparts. The same yeast strain was cultured and treated with or without VPA for 5 min. WCEs were collected, a portion of which was treated with phosphatase for 1 h. Samples were then electrophoresed on a Zn^2+^ Phos-tag gel and analyzed by WB. To confirm that all the bands observed were tagged MIPS, WT cells expressing untagged MIPS were also cultured and treated in parallel. This control validates the specificity of the antibody, confirming that the shifted bands in the 6xHis-Xpress-MIPS-containing lanes are tagged MIPS. 6xHis-Xpress-MIPS exhibited two separate bands of lower mobility (bands appearing higher on the membrane relative to MIPS), only one of which disappeared with phosphatase treatment. The band that disappeared with phosphatase treatment was considered to be phosphorylated MIPS. The results indicate that even without VPA treatment, a small amount of phosphorylated MIPS (≈ 0.8% of total) was detected (Fig. 1b). Treatment with VPA induced only a small increase in the amount of phosphorylated MIPS (≈ 1.3% of total). Even factoring in the lower mobility band that did not disappear with phosphatase treatment, less than 2% of MIPS was phosphorylated. It is worth noting that the plasmid expression levels of 6xHis-Xpress-MIPS from pADH-*INO1* were not greater than endogenous expression levels of MIPS (Fig. 3 WT vs. ino1∆ + pADH-*INO1*), so the relevant amount of phosphorylation is not augmented as a result of overexpression from a plasmid. The low level of observed phosphorylation suggests that it may not be a significant means of regulating overall MIPS activity, as > 98% remains unphosphorylated.

### Identification of protein kinases that phosphorylate MIPS

In an attempt to identify the regulatory protein kinase of MIPS, we employed two different screens, both of which have been demonstrated to identify transient interactions. The first is the in vivo protein–protein interaction biotin identification (BioID) screen, which utilized a promiscuous biotin ligase (BioID2 in this study)^28^ fused to MIPS. This fused protein, as well as the unfused BioID2 control, was expressed from plasmids in *ino1*∆ cells. Cultures were treated with or without VPA for 1 h, allowing time for interacting, biotinylated proteins to accumulate. The biotinylated proteins were then purified from WCEs and subjected to liquid chromatography—tandem MS (LC–MS/MS) proteomic analysis. As expected, naturally biotinylated carboxylases^29,30^ were among the most abundant proteins identified in all samples; however, the strains expressing the fused construct (BioID2-MIPS) had enhanced labeling and/or enrichment of these carboxylases relative to the unfused BioID2 control. Due to this discrepancy, the MS quantities were normalized to the enrichment of carboxylases in the sample. While these natively biotinylated carboxylases were relatively abundant in the samples, very few proteins were enriched in the BioID2-MIPS samples (Table 1; see Table S1 online for complete list). One explanation for the low abundance of interacting proteins is that MIPS does not readily associate with other proteins, and the proteins identified possibly result from non-specific interactions. Mck1 was the most enriched protein kinase identified in the screen, but at a very low abundance. The addition of VPA did not enhance any protein kinase interactions with MIPS.

**Table 1:** BioID screen enrichment analysis: MIPS interacting proteins.

Accession	Description	1	0.559	0.154	0.137	← Normalization factors		
		BioID2-MIPS − VPA total (normalized to carboxylases)	BioID2-MIPS + VPA total (normalized to carboxylases)	Unfused BioID2 − VPA total (normalized to carboxylases)	Unfused BioID2 + VPA total (normalized to carboxylases)	BioID2-MIPS − VPA/unfused BioID2 − VPA enrichment	BioID2-MIPS + VPA/unfused BioID2 + VPA enrichment	+ VPA/ − VPA enrichment
*ACAC*	*Acetyl-CoA carboxylase*	*942*	*1034*	*572*	*576*	*1.65*	*1.80*	*1.09*
*PYC1*	*Pyruvate carboxylase 1*	*474*	*431*	*611*	*612*	*0.78*	*0.70*	*0.91*
*PYC2*	*Pyruvate carboxylase 2*	*457*	*408*	*689*	*685*	*0.66*	*0.60*	*0.90*
DUR1	Urea amidolyase	301	247	325	335	0.93	0.74	0.80
ARC1	tRNA-aminoacylation cofactor ARC1	247	297	976	743	0.25	0.40	1.58
INO1	Inositol-3-phosphate synthase	65	88	0	0	Enriched	Enriched	N/A
COPE	Coatomer subunit epsilon	35	20	78	66	0.45	0.30	0.67
*HFA1*	*Acetyl-CoA carboxylase mitochondrial*	*35*	*13*	*0*	*0*	*Enriched*	*Enriched*	*N/A*
ABP1	Actin-binding protein	20	0	7	36	3.08	N/A	N/A
RPA43	DNA-directed RNA polymerase I subunit RPA43	17	0	26	51	0.65	N/A	N/A
SYRC	Arginine–tRNA ligase cytoplasmic	4	2	0	0	Enriched	Enriched	N/A
SYEC	Glutamate–tRNA ligase cytoplasmic	3	2	0	0	Enriched	Enriched	N/A
G3P1	Glyceraldehyde-3-phosphate dehydrogenase 1	3	0	52	15	0.06	N/A	N/A
G3P2	Glyceraldehyde-3-phosphate dehydrogenase 2	3	5	52	15	0.06	0.37	6.39
G3P3	Glyceraldehyde-3-phosphate dehydrogenase 3	3	5	52	15	0.06	0.37	6.39
**MCK1**	**Protein kinase MCK1**	**2**	**0**	**0**	**0**	**enriched**	**N/A**	**N/A**

Yeast cells expressing either BioID2-MIPS or BioID2 unfused control were treated with or without VPA (1 mM) with excess biotin (n = 4). Biotin labeled proteins were purified from WCEs with magnetic streptavidin beads and identified by MS proteomic analysis. MS spectral counts for each protein identified were totaled from the replicates of each sample type then normalized to the carboxylases in the sample (italics), which are naturally biotinylated and account for variations of strain biotinylation activity and streptavidin purification efficiency. A fold enrichment analysis was performed to determine MIPS specific interactions (BioID2-MIPS/unfused BioID) with or without VPA treatments. These were then used to determine VPA-induced interactions (+ VPA/− VPA). “enriched” indicates incalculable enrichments (due to an unfused BioID2 spectral count of zero) and “N/A” indicates incalculable non-enrichments (due to a MIPS-BioID2 spectral count of zero). Kinases are indicated with bold font. See Supplementary Table S1 for raw spectral count data and complete table with calculation.

**Figure 2:**
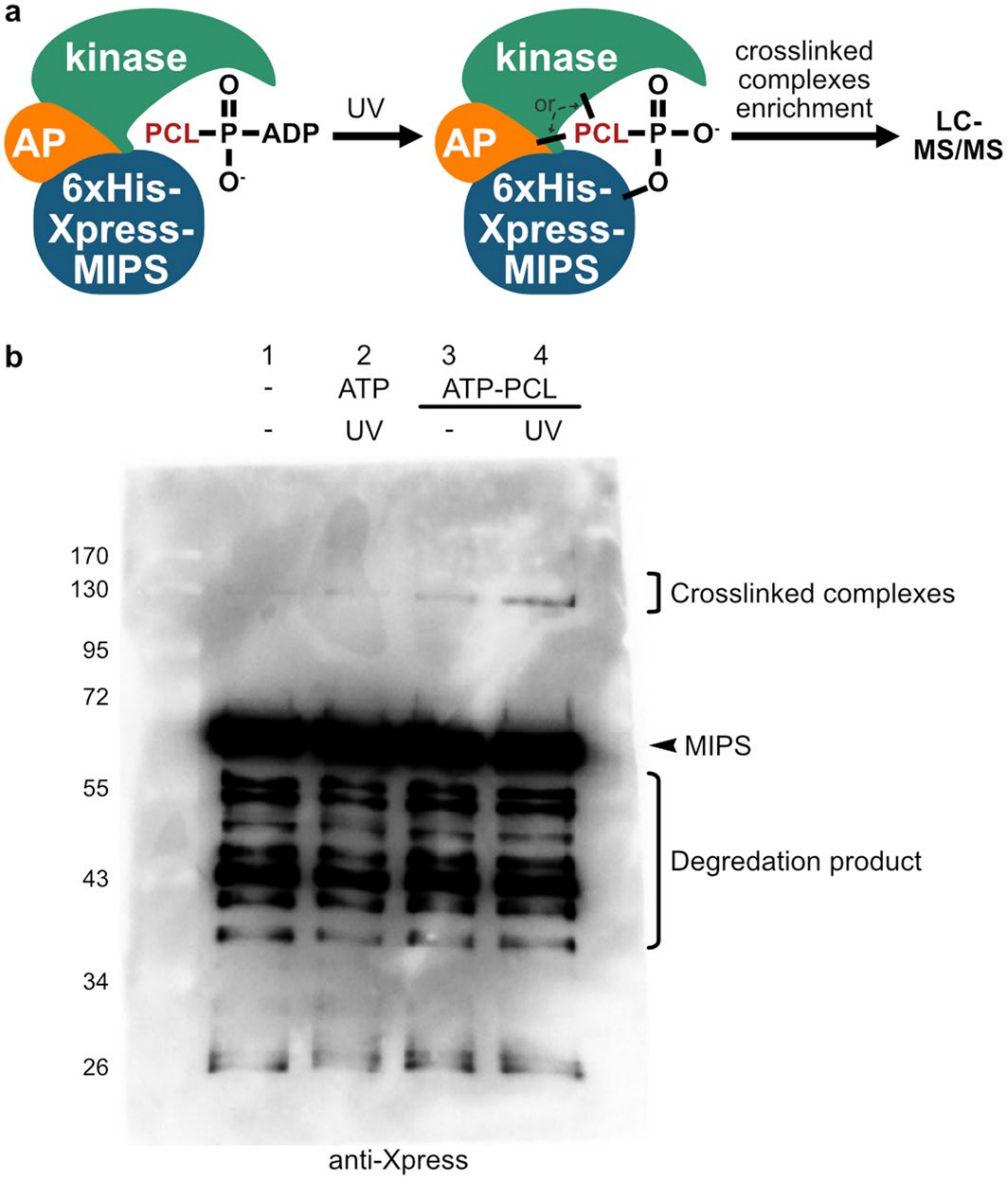
K-CLiP screen: identification of MIPS interacting protein kinases and associated proteins. (**a**) Protein kinase uses the γ-phosphoryl-modified ATP-photocrosslinker (ATP-PCL) analogue, ATP-arylazide, as a cosubstrate to transfer the arylazide photocrossinker to 6xHis-Xpress-MIPS. Under UV irradiation, the photocrosslinker covalently attaches to the kinase or associated proteins (APs). The crosslinked complexes are enriched by 6xHis purification. (**b**) WCEs containing 6xHis-Xpress-MIPS (1.5 mg total protein) were incubated with or without ATP or ATP-PCL and UV irradiation (n = 2). MIPS and MIPS-crosslinked complexes were isolated by nickel magnetic beads and a portion (1/3 of the reaction) was analyzed by SDS-PAGE WB (shown is one replicate) to confirm photocrosslinking. 6xHis-Xpress-MIPS crosslinked complexes have a higher molecular weight relative to 6xHis-Xpress-MIPS alone.

The second screen involved covalently crosslinking kinases to their substrates in a kinase-dependent manner, followed by identification of the crosslinked pairs by LC–MS/MS analysis. The kinase-catalyzed crosslinking and ion purification (K-CLiP) method^31^ uses a γ-phosphoryl-modified adenosine 5’-triphosphate (ATP) analogue containing a photocrosslinking group to covalently link MIPS to both kinases and associated proteins. In K-CLiP, the ATP-photocrosslinker (ATP-PCL), ATP-arylazide, acts as a co-substrate of cellular kinases to transfer the arylazide photocrosslinker to 6xHis-Xpress-MIPS via kinase activity^32^. In the presence of UV irradiation, which activates the photoreactive arylazide, a covalent crosslink can form between MIPS and kinase(s) or associated proteins, and the complexes can be subsequently enriched and characterized (Fig. 2a). WCEs prepared from *ino1*∆ + pADH-*INO1* were incubated with ATP-PCL and UV irradiation alongside control reactions to determine crosslinking efficiency and identify proteins crosslinked to MIPS. 6xHis-Xpress-MIPS, along with crosslinked proteins, were then isolated by nickel ion beads and a portion was utilized for WB analysis to confirm crosslinking. Only in the presence of both ATP-PCL and UV did we observe an increase in the amount of high molecular weight crosslinked complexes containing 6xHis-Xpress-MIPS (Fig. 2b, lane 4) compared to the controls (Fig. 2b, lanes 1–3). The isolated crosslinked complexes were subjected to LC–MS/MS and MaxQuant analyses^33^ to identify the MIPS-kinase and MIPS-associated protein pairs. An enrichment analysis consolidated the list of identified proteins to reveal those most likely to interact with MIPS (Table 2; see Table S2 online for complete list and Table S3 online for raw data). The two most enriched protein kinases were *AKL1* and *PTK2,* which were investigated further.

**Table 2:** K-CLiP screen enrichment analysis: MIPS interacting kinases and associated proteins.

Protein name	Gene	Trial 1 fold change	Trial 2 fold change
**Serine/threonine-protein kinase AKL1**	**AKL1**	**∞**	**∞**
60S ribosomal protein L37-A	RPL37A	∞	∞
Heat shock protein SSA2	SSA2	∞	∞
Translationally-controlled tumor protein homolog	TMA19	∞	∞
Cerevisin	PRB1	12	∞
Saccharopepsin	PEP4	∞	8
Exocyst complex component EXO70	EXO70	∞	7
**Serine/threonine-protein kinase PTK2/STK2**	**PTK2**	2	**∞**
RNA-binding protein SGN1	SGN1	31	3
40S ribosomal protein S11-B;40S ribosomal protein S11-A	RPS11B; RPS11A	9	16
60S ribosomal protein L2-B;60S ribosomal protein L2-A	RPL2B; RPL2A	6	3
Protein PAL1	PAL1	4	4
60S ribosomal protein L28	RPL28	4	3
Protein HBT1	HBT1	2	3
Heat shock protein SSA1	SSA1	2	2

WCEs containing 6xHis-Xpress-MIPS were incubated with or without ATP or ATP-PCL and UV irradiation (n = 2). Proteins were isolated by nickel magnetic beads and analyzed by LC–MS/MS proteomic analysis. Protein enrichment analyses using label-free quantification by MaxQuant were performed by first removing background protein (proteins nonspecifically bound to the nickel beads in WCE only control). Second, the proteins that were enriched greater than or equal to two-fold in the ATP-PCL with UV irradiation samples relative to the ATP-PCL without UV irradiation samples in both trials are shown in the hit list. Kinases are indicated with bold font. Fold enrichments labeled with “**∞**” identifies proteins where the peak intensities of the background controls (WCE only and ATP-PCL without UV) were zero, which indicates infinite enrichment. See Supplementary Table S2 for complete enrichment analysis and Supplementary Table S3 for raw data.

### Only tagged MIPS is phosphorylated in vivo

**Figure 3:**
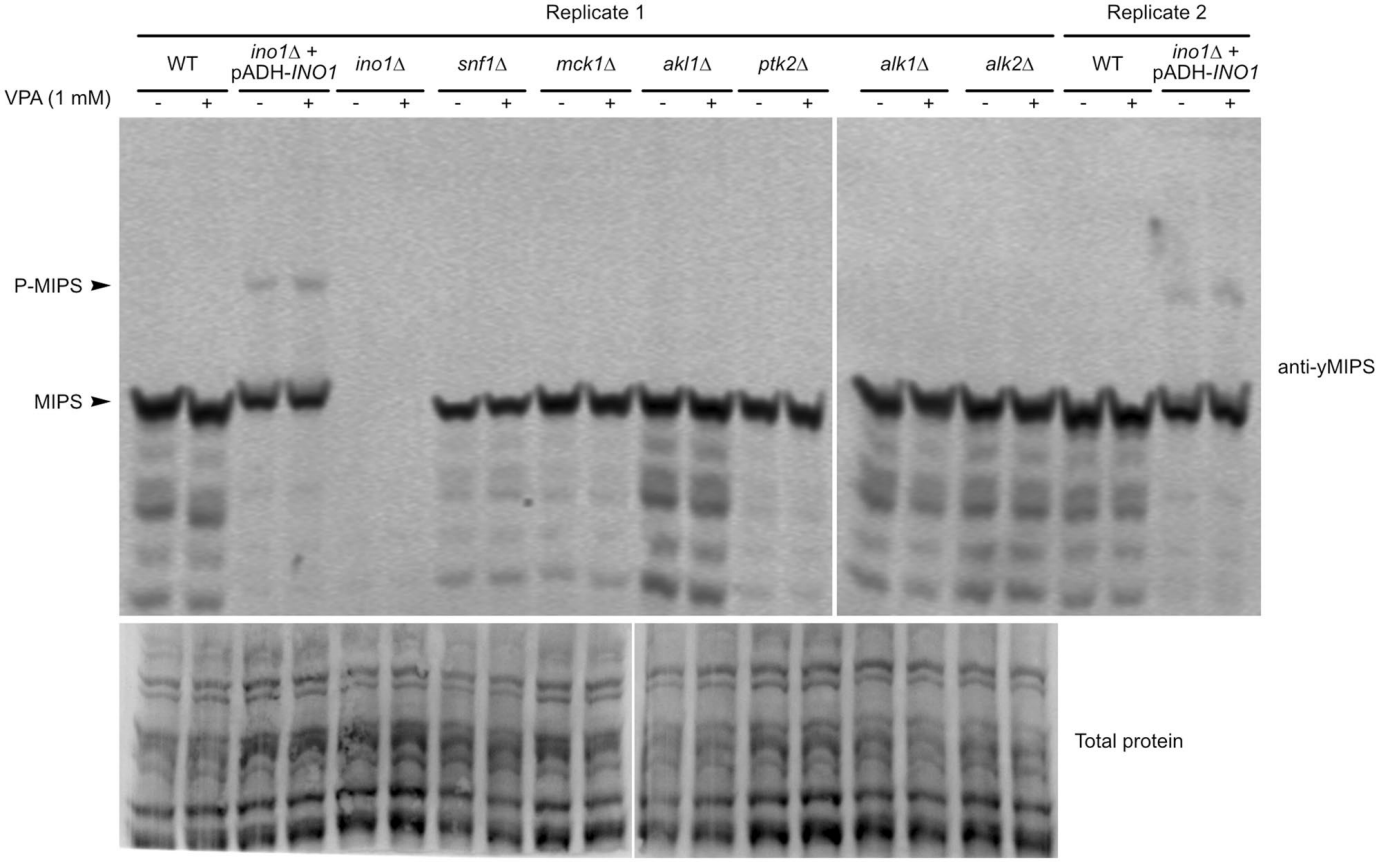
Only 6xHis-Xpress tagged MIPS is phosphorylated in vivo. Cultures of WT, *ino1*∆ + pADH-*INO1*, *ino1*∆, and protein kinase deletion mutants were treated with or without 1 mM VPA for 5 min then WCEs (20 µg total protein) were analyzed by Zn^2+^ Phos-tag WB using an antibody specific for yeast MIPS. MIPS is absent from *ino1*∆ lysates which controls for antibody specificity, confirming that the observed bands are specific to MIPS. A second independent experiment of WT and *ino1*∆ + pADH-*INO1* was also assayed to confirm results. To show similar protein quantities between samples, replicate 1 was separately assayed by Zn^2+^ Phos-tag WB and total protein labeled (a separate WB was necessary as we suspected the two imaging techniques may be incompatible). Phosphorylated proteins migrate slower than their unphosphorylated versions. The lower molecular weight bands below the MIPS bands are likely degradation by-products and a few faint non-specific bands. See uncropped/unedited membrane image in Supplementary Fig. S2 online.

The most abundant protein kinases from the two screens that potentially interact with MIPS included *AKL1*, *PTK2*, and *MCK1*. Surprisingly, none of the kinases identified in the BioID analysis were also identified in the K-CLiP screen; the closest match included the paralogs *AKL1* (K-CLiP) and *AKL2* (BioID), both of which ranked low in the interaction tables (see Tables S1 and S2 online for full lists of identified proteins). *SNF1* (the yeast AMPK homolog, a major metabolic regulator) was also tested as a putative kinase because Snf1 itself as well as other subunits of the protein kinase complex (*SNF4*, *SIP2*, and *GAL83*) were found in low abundance in the K-CLiP screen. Furthermore, Snf1 (or AMPK) is activated by VPA in both yeast^34–36^ and mammalian cells^4,37^. To determine if these protein kinases can lead to MIPS phosphorylation, we utilized kinase deletion strains and tested for phosphorylation of endogenously expressed MIPS with VPA treatment using Zn^2+^ Phos-tag WB analyses and an antibody that can interact with yeast MIPS (anti-yMIPS) (Fig. 3). The *ino1*∆ + pADH-*INO1* strain (expressing 6xHis-Xpress-MIPS) was used as a control, as we were previously able to detect reduced mobility caused by phosphorylation (Fig. 1b). Use of this strain also confirms that the antibody is capable of interacting with phosphorylated MIPS. Surprisingly, MIPS was not phosphorylated in any of the strains expressing untagged, endogenously expressed MIPS. This finding suggested that the presence of the tag led to an increase in the amount of observed phosphorylation. Either phosphorylation of 6xHis-Xpress-MIPS occurs on the tag and not within the peptide sequence of MIPS itself, or the presence of the tag induces a protein kinase interaction with MIPS. Nonetheless, the results show a lack of phosphorylation of untagged MIPS, suggesting that the observed phosphorylation is exaggerated by the presence of the 6xHis-Xpress tag.

### PKC and CKII in vitro phosphorylation of MIPS

**Figure 4:**
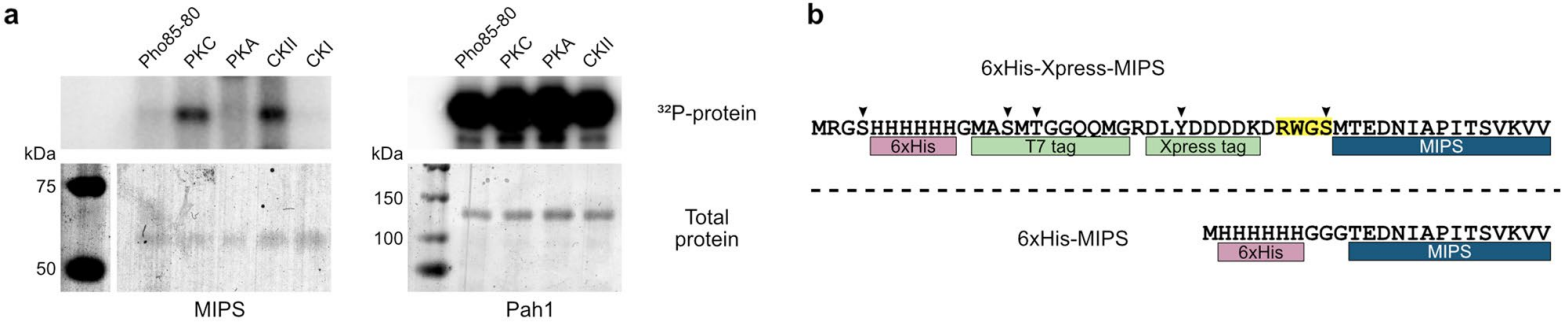
In vitro phosphorylation of tagged MIPS. (**a**) PKC and CKII phosphorylate 6xHis-Xpress-MIPS in vitro*.* Purified 6xHis-Xpress-MIPS was incubated with purified protein kinases in the presence of radioactive [γ-^32^P]ATP. Control reactions to confirm kinase activity utilized 6xHis-Pah1 as the substrate. Reactions were then run on SDS-PAGE gels and phosphorylation imaged by autoradiography (top) and total protein stained by Coomassie blue (bottom) (n = 2; shown is a representative result, see uncropped images in Supplementary Fig. S3 online) (**b**) Amino acid sequences of 6xHis-Xpress tagged MIPS (top) and the short-tagged 6xHis-MIPS (bottom). Arrows indicate amino acids potentially phosphorylated in yeast and a PKC consensus sequence motif (R*XX*S) is highlighted. For reference, the N-terminal sequence of yeast MIPS is shown.

To address the possibility that MIPS phosphorylation was not observed as a result of loss of phosphorylation during cell lysis, we carried out in vitro analyses of MIPS phosphorylation. Candidate kinases PKC, PKA, CKII, CKI, and Pho85-80 were tested for their ability to phosphorylate MIPS. PKA and PKC were predicted by NetPhos Yeast to phosphorylate the putative phosphosites identified previously^19^. This was corroborated by performing a motif sequence prediction of the full-length yeast and human MIPS protein sequences using Scansite 4.0^38^ and NetPhos 3.1^39^ (data not shown). CKII was also predicted based on the sequence motif. The candidate kinase screen revealed that 6xHis-Xpress-MIPS can be phosphorylated in vitro by PKC (mixture of α, β, and γ from rat brain) and human CKII (Fig. 4a), whereas the control Pah1 substrate was phosphorylated by Pho85-80^40^, PKC^41^, PKA^42^, and CKII^43^, as expected.

**Figure 5:**
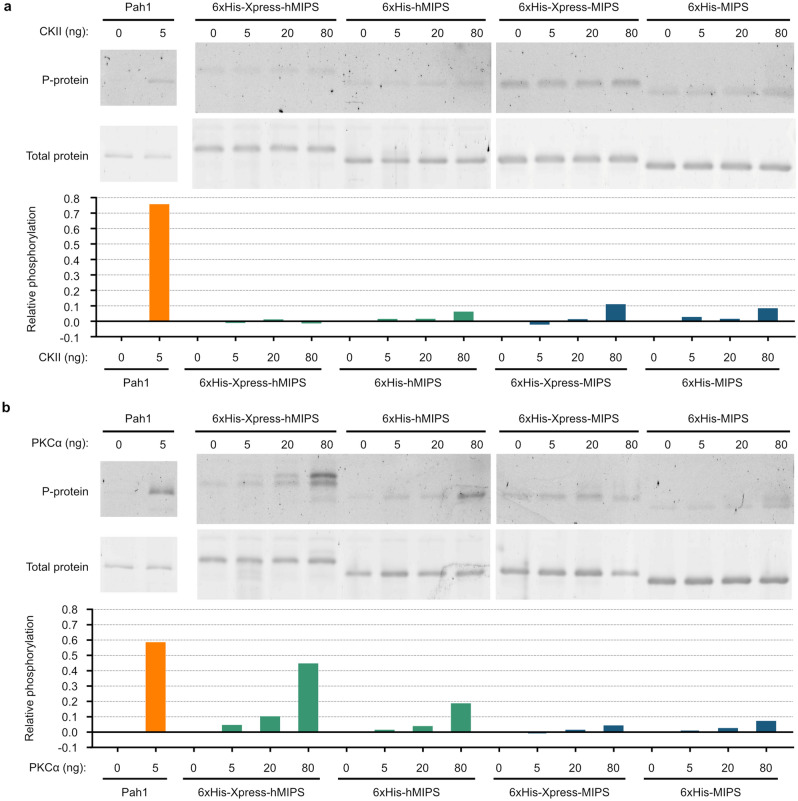
In vitro phosphorylation analyses of different tagged versions of MIPS. Two differently tagged versions of human and yeast MIPS (hMIPS and MIPS, respectively), one containing the Xpress epitope and amino acids capable of being phosphorylated in yeast (6xHis-Xpress) and the other lacking these (6xHis) (see Fig. 4), were incubated with ATP and increasing amounts of CKII (**a**) and PKCα (**b**). Pah1 was used as a substrate control to confirm kinase activity. Reactions were then assayed by SDS-PAGE and phosphorylated proteins stained with Pro-Q Diamond followed by total protein staining with SYPRO Ruby (see uncropped/unedited images in Supplementary Fig. S5 online). Relative phosphorylation quantifications are shown below their respective gel staining analyses. As Pro-Q Diamond phosphoprotein stain has some innate affinity to MIPS, the band density from the reactions with 0 ng kinase was subtracted as background from the other band density quantitations of that reaction group.

As discussed, in vivo MIPS phosphorylation could only be detected if MIPS was tagged with 6xHis-Xpress. The sequence of the tag includes five amino acids that can be phosphorylated in yeast (serine, threonine, and tyrosine), one of which is within a PKC consensus sequence motif (Fig. 4b). LC–MS/MS proteomic analysis of 6xHis-Xpress-MIPS identified phosphorylation at this PKC motif (S36 of the 6xHis-Xpress epitope), and at the N-terminus of MIPS (S43) (Supplementary Fig. S4 online). We therefore predicted that the in vitro phosphorylation we observed may be isolated to the tag. To test this possibility, four plasmids were constructed to express 6xHis-Xpress tagged human and yeast MIPS (6xHis-Xpress-hMIPS and 6xHis-Xpress-MIPS) in addition to short-tagged versions, which lack amino acids on the tag that can be phosphorylated in yeast (6xHis-hMIPS and 6xHis-MIPS) (Fig. 4b). The tagged MIPS proteins were purified from yeast and used as substrates for CKII and PKC in optimized reaction conditions. Pah1 was used as a substrate control to confirm that kinases were active. MIPS phosphorylation did not increase with increasing amounts of CKII, regardless of the presence of the tag (Fig. 5a), indicating that the reaction is likely non-specific. In contrast, phosphorylation of MIPS was slightly greater with PKCα (Fig. 5b). The longer tagged version of hMIPS had twice as much phosphorylation relative to the short-tagged hMIPS substrate, suggesting that PKCα phosphorylates the 6xHis-Xpress tag. However, eliminating this tag did not completely abolish phosphorylation, suggesting that PKCα can phosphorylate MIPS to a limited degree. To determine if PKCα phosphorylation of MIPS (human and yeast) is specific, we analyzed the kinetics of the reaction. Quantification of the amount of phosphorylated MIPS in response to varying time, ATP, and substrate (MIPS) produced relatively flat curves with no defined plateau, or a linear curve in the case of increasing PKC (Supplementary Fig. S6 online), but not bell-shaped curves which are expected if the reactions were specific. These findings suggest that the specific activity of MIPS phosphorylation by PKCα is low.

### MIPS phosphorylation is not observed in response to VPA when the 6xHis-Xpress tag is absent

**Figure 6:**
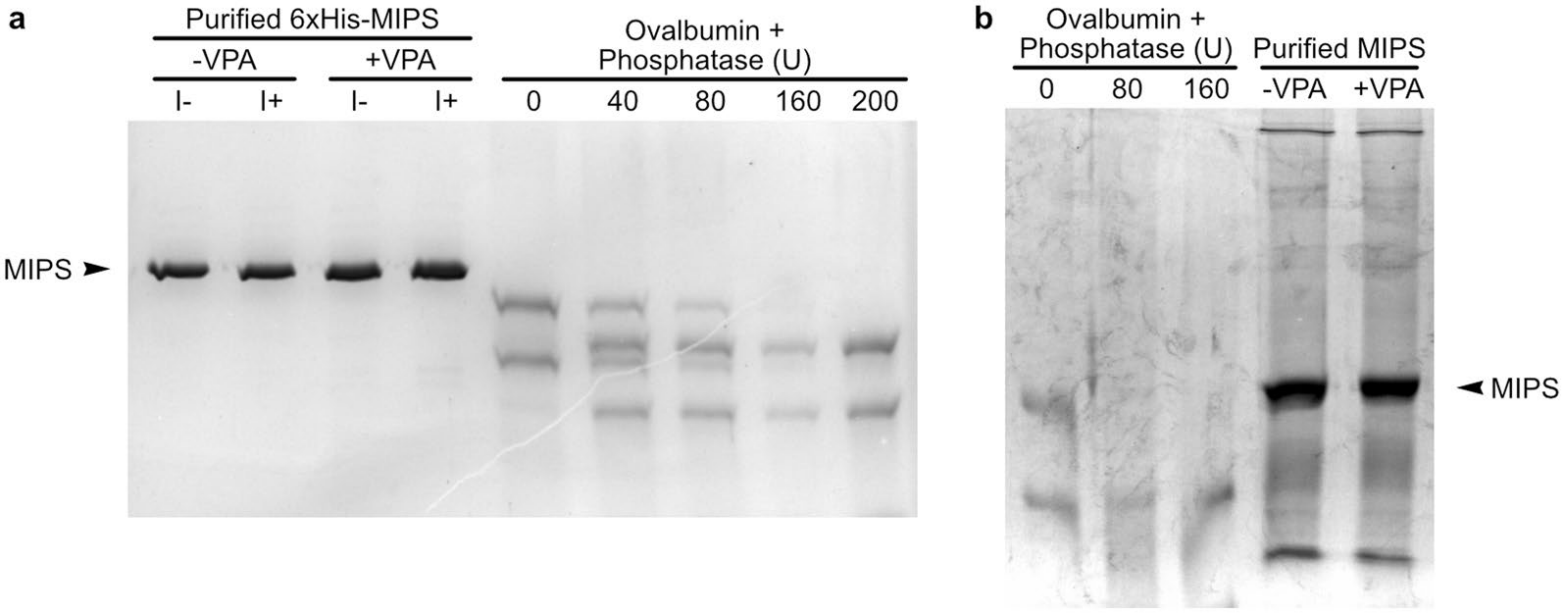
In vivo phosphorylation of MIPS in response to VPA treatment is undetectable in the absence of the 6xHis-Xpress tag. (**a**) Yeast cells overexpressing short-tagged 6xHis-MIPS from a vector were treated with or without 1 mM VPA for 1 h in the presence or absence of inositol. 6xHis-MIPS was isolated from WCEs with Ni–NTA resin by batch purification then concentrated. Phosphorylation of purified proteins (0.5 µg) was determined by Mn^2+^ Phos-tag SDS-PAGE analysis and proteins stained with Coomassie blue. Phosphorylated albumin (0.5 µg) from chicken egg whites (ovalbumin) was treated with increasing amounts of phosphatase and assayed simultaneously to verify Phos-tag gel efficacy. (**b**) Cultures of WT yeast were treated with or without 1 mM VPA for 1 h, then endogenously expressed MIPS was immunoprecipitated from WCEs with anti-yMIPS antibodies. Phosphorylation of purified proteins was assayed as before. Phosphorylated proteins migrate slower than their unphosphorylated versions in Phos-tag gels. See uncropped gel images in Supplementary Fig. S7 online.

The experiments described above indicated that the 6xHis-Xpress tagged MIPS is phosphorylated in vivo and in vitro*.* We next wished to determine the fraction of untagged MIPS that is phosphorylated in vivo in response to VPA. Due to the technical challenges and potential pitfalls of Phos-tag WB analysis (such as low transfer efficiency of phosphorylated proteins to the membrane, or low interactions of certain phospho-groups with the ionized Phos-tag acrylamide in the gel), we implemented in-gel total protein staining of Mn^2+^ Phos-tag gels (to eliminate the pitfall of low transfer efficiency). To do so, we utilized a yeast strain expressing short-tagged yeast MIPS (6xHis-MIPS) from a high-expression vector (*ino1*∆ + pyPGK18-6xHis-MIPS), which lacks amino acids on the tag that can be phosphorylated in yeast. The cultures were treated with or without VPA in the presence or absence of inositol. 6xHis-MIPS was then isolated from WCEs and concentrated to a very high level of purity. The proteins were analyzed by Mn^2+^ Phos-tag SDS-PAGE and Coomassie blue total protein staining. A highly phosphorylated protein, albumin from egg whites (ovalbumin), was treated with increasing amounts of phosphatase to authenticate the reduced mobility of phosphorylated, relative to non-phosphorylated, proteins in the Phos-tag gels. Similar to earlier results (Fig. 3), no phospho-protein shift was observed for any of the 6xHis-MIPS proteins (Fig. 6a). To address the possibility that the lack of phosphorylation was due to overexpression of MIPS, we used the same analytical approach to examine endogenously expressed MIPS purified utilizing anti-yMIPS antibodies. As endogenously expressed MIPS is transcriptionally repressed when grown in the presence of inositol, this analysis was performed in its absence. Although the purified protein was not enriched to the same degree as 6xHis-MIPS, the results similarly showed no observable phosphorylation of MIPS isolated from either VPA-treated or -untreated cells (Fig. 6b). These findings suggested that MIPS is unlikely phosphorylated to a biologically significant level in response to VPA treatment.

### VPA reduces MIPS expression

While inositol depletion is a well-established outcome of VPA treatment ^19–23^, the mechanism by which this occurs has remained elusive. However, there is a consensus that the mechanism ultimately leads to reduced MIPS enzymatic activity^19,20,22,23,27^. As phosphorylation of MIPS appears to be an unlikely mechanism of reducing inositol synthesis, we hypothesized that VPA-induced inositol depletion may be due to decreased expression of MIPS. Typically, when yeast cells are transferred to fresh glucose medium lacking inositol, *INO1* expression rapidly increases and is highest during the mid-log growth phase^20^. As cells enter the late-log/early-stationary growth phase, expression is repressed. Interestingly, the expression profile of *INO1* is altered when yeast cells are grown in the presence of VPA as *INO1* levels were reduced during mid-log phase but increased during stationary phase^20^.

**Figure 7:**
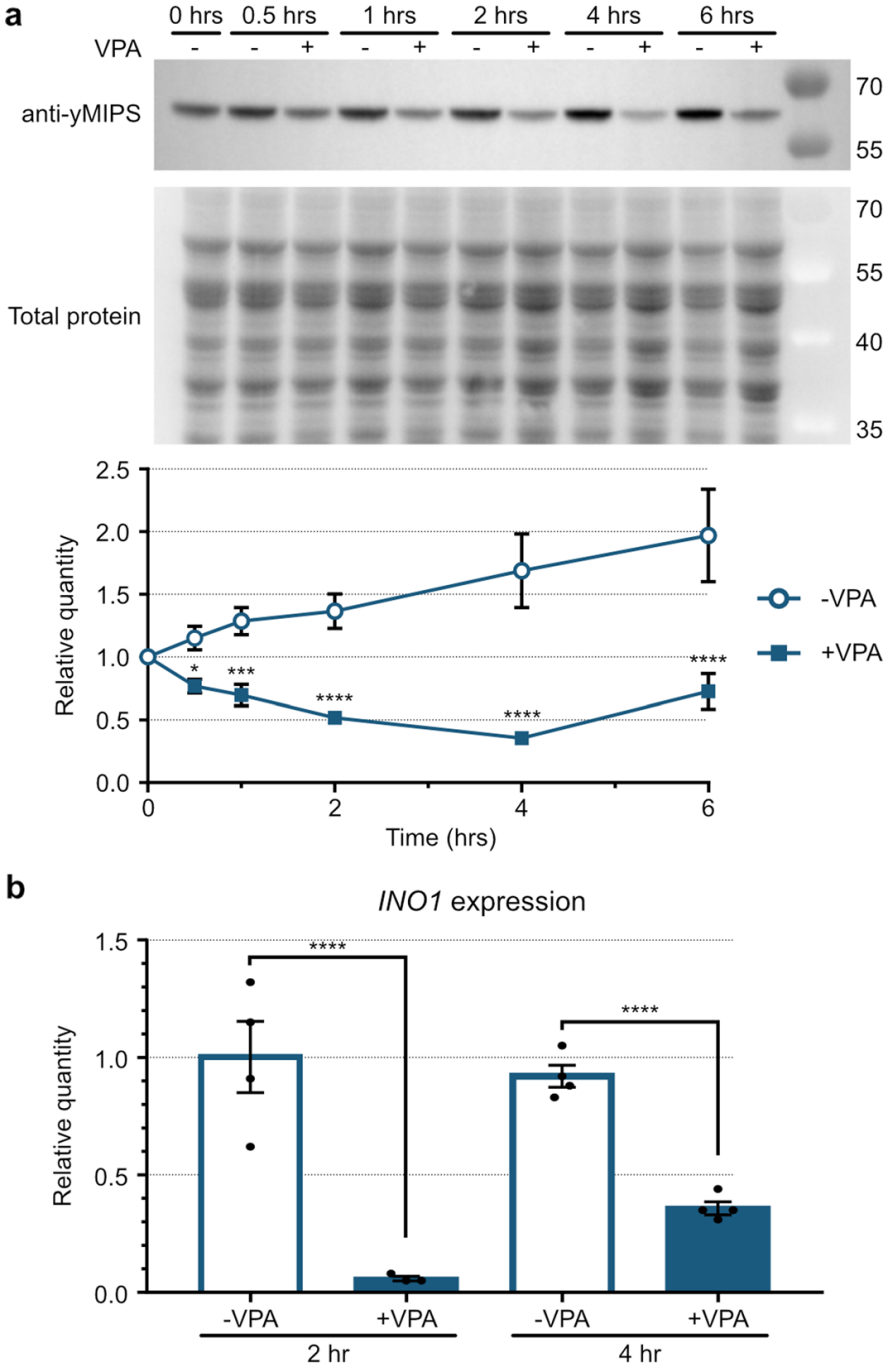
MIPS expression is reduced upon VPA treatment. (**a**) Yeast cells grown in the absence of inositol to the early-log growth phase were treated with or without 1 mM VPA for up to 6 h (n = 3). WCEs were analyzed by SDS-PAGE and WB using anti-yMIPS antibodies and a protein labeling reagent to detect total protein (top; representative of three replicates, see uncropped images in Supplementary Fig. S8 online). Bands were quantified and MIPS was normalized to total protein (bottom; SD, two-way ANOVA). (**b**) RT-qPCR analysis of *INO1* mRNA levels from yeast grown in the absence of inositol and treated with or without 1 mM VPA for 2 and 4 h (n = 4; SEM, ordinary one-way ANOVA). *p < 0.05, **p < 0.01, ***p < 0.001, ****p < 0.0001.

Utilizing our anti-yMIPS antibody and focusing on the effects of VPA during the mid-log growth phase, we examined MIPS protein levels after 30 min and up to 6 h of VPA treatment. VPA significantly decreased MIPS protein levels relative to untreated yeast within 30 min (≈ 30% decreased) and this difference grew at later times (≈ 60% decreased) (Fig. 7a). In accordance with this finding, *INO1* expression was also significantly reduced after 2 and 4 h of VPA treatment (Fig. 7b). These data suggest that the mechanism of VPA-induced inositol depletion is via decreased expression of *INO1* during the mid-log growth phase, ultimately decreasing MIPS protein levels and inositol synthesis.

## Discussion

Previous studies utilizing a yeast strain expressing a 6xHis-Xpress tagged MIPS demonstrated decreased activity of 6xHis-Xpress-MIPS purified from cells treated with VPA, which was increased by incubation with alkaline phosphatase. Phosphosites were identified by MS proteomic analysis and results from site-directed mutagenesis of these sites indicated that phosphorylation alters enzymatic activity, suggesting that VPA inhibits MIPS by inducing phosphorylation^19^. This hypothesis predicts that a significant pool of MIPS must be phosphorylated to reduce overall activity. To test this, we carried out experiments to determine if significant levels of MIPS phosphorylation were induced by VPA. The findings reported here demonstrate that the pool of phosphorylated 6xHis-Xpress-MIPS is less than 2% of total MIPS. Removal of the epitope tag reduced the amount of phosphorylated MIPS to undetectable levels. This was revealed by implementation of a custom antibody directed against amino acids 348 – 370 of the yeast MIPS protein (commercially available antibodies purported to recognize the yeast isoform did not exhibit specificity for the enzyme). Thus, the presence of the tag artificially elevated the fraction of phosphorylated MIPS both in vivo and with in vitro kinase incubation. One potential explanation for this was that the 6xHis-Xpress epitope is phosphorylated. In support of this, the 6xHis-Xpress tag has amino acids that can be phosphorylated in yeast, one of which is within a PKC consensus sequence. LC–MS/MS proteomic analysis of 6xHis-Xpress-MIPS did indeed find this PKC motif to be phosphorylated. Removal of this epitope reduced phosphorylation of hMIPS by PKCα by half, indicating that PKCα may phosphorylate this tag. As the amount of phosphorylated MIPS was very low (< 2% of 6xHis-Xpress-MIPS and undetectable levels for untagged MIPS), it is unlikely that phosphorylation regulates overall MIPS activity levels in response to VPA. Consistent with this, the two different screens used to identify MIPS-interacting proteins identified few protein kinases and no kinases were commonly identified by both screens. We posit that most of the interactions captured could be due to either infrequent or low-specificity interactions. This is illustrated by the in vitro phosphorylation reactions with CKII and PKCα, both of which have low-activity for MIPS and lead to low levels of phosphorylation, likely a consequence of the promiscuous activity of these kinases. In conjunction with these findings, we did not observe phosphorylation of untagged MIPS from cells treated with VPA, arguing against the hypothesis that VPA inhibits MIPS activity by inducing phosphorylation^19^.

How then does VPA lead to inhibition of MIPS and inositol depletion? Our current findings suggest that MIPS activity is reduced in response to VPA treatment by inhibiting expression of the MIPS encoding gene, *INO1,* resulting in a concomitant decrease of MIPS protein. We show that VPA reduces *INO1* mRNA levels during the logarithmic growth phase, consistent with a previous report^20^. Utilizing the custom yMIPS antibody, we now show that this decrease in *INO1* mRNA expression is reflected in decreased MIPS protein levels. This novel finding offers a more likely explanation for the reduced rate of inositol synthesis observed in yeast cells in response to VPA.

Taken together, these data indicate that VPA causes inositol depletion by reducing MIPS expression rather than by inducing phosphorylation of the protein. Nevertheless, we cannot rule out the possibility of a transient kinase interaction that may have been missed with the approaches taken, although the screens for interacting kinases were specifically optimized for capturing these types of interactions. Furthermore, the in vitro candidate kinase assays screened only five kinases, while yeast have potentially over a hundred kinases (see yeastkenome.org). In addition, the in vitro reactions may not replicate the in vivo conditions necessary for specific phosphorylation of MIPS, even if the kinases are active under our experimental parameters. Importantly, it is possible that physiological conditions not tested lead to phosphorylation of the protein. Finally, whether MIPS is phosphorylated in other species remains to be determined. Mammalian MIPS (encoded by *ISYNA1*) exhibits some structural dissimilarities with the yeast enzyme. Specifically, some isoforms contain a longer C-terminal tail. Phosphosites on this tail region have been identified in rats by proteomic analyses^44^ but lack sequence similarity to yeast MIPS. Whether these isoforms are regulated by phosphorylation remains to be determined. Finally, MIPS purified from yeast treated with VPA consistently exhibits decreased enzymatic activity relative to MIPS from untreated cells^19^ (and data not shown), suggesting that VPA may induce some modifications of MIPS other than phosphorylation. Whether this decrease in enzymatic activity contributes to the inositol depletion effects of VPA remains in question, especially as we demonstrated a significant decrease in MIPS enzyme levels under these conditions which could explain the decrease in inositol levels observed following VPA treatment. While the current findings indicate that VPA leads to reduced expression of MIPS during the mid-log growth phase, the mechanism underlying this effect is not understood. Studies to uncover this mechanism are currently underway.

## Methods

### Yeast strains, medium, and growth conditions

*S. cerevisiae* yeast strains used in the study were BY4741 WT and derived deletion mutants *ino1*∆, *snf1*∆, *mck1*∆, *akl1*∆, *ptk2*∆, *alk1*∆, and *alk2*∆ (replaced with KanMX4) from Invitrogen. Mutants were confirmed by PCR. Cells were maintained on YPD medium (2% glucose, 2% bactopeptone, 1% yeast extract, 2% agar) and cultured in synthetic complete 2% glucose (SCD) medium without inositol (I-), containing adenine (4 mg/L), arginine (4 mg/L), histidine (2 mg/L), leucine (12 mg/L), lysine (4 mg/L), methionine (4 mg/L), threonine (60 mg/L), tryptophan (4 mg/L), uracil (8 mg/L), and all components of Wickerham’s yeast nitrogen base (except inositol). Inositol (75 µM) was added where indicated (I+). Cell culture ocular densities (OD) were measured in a spectrophotometer with an wavelengh of 600 nm (OD_600_). Cells were inoculated in medium (5–10 mL) and incubated overnight (preculture), washed in 20 mL sterile water by pelleting cells by centrifugation at 3000 × g for 5 min, discarding supernatant, vortexing in water, and centrifugation repeated to collect cell pellet. Washed cells were then suspended in sterile water (5–10 mL) prior to inoculating fresh medium for experimentation. All incubations were at 30 °C with shaking (230 rpm) for liquid cultures. All cultures were in mid-log phase of growth when VPA was administered.

### Plasmid construction and yeast transformation

BioID2 expression plasmids were cloned using the In-Fusion^®^ HD cloning kit according to the user manual. Primers for PCR amplifying the myc-BioID2 fragment from the myc-BioID2-MCS and mycBioID2-13X Linker-MCS plasmids ^28^ were designed using the SnapGene In-Fusion^®^ tool. The pADH-*INO1* vector^19^ was linearized with XbaI and BamHI restriction enzymes (Promega). Both PCR fragments and linearized vector were purified using the NucleoSpin^®^ Gel and PCR Clean-up kit (Takara). The fragment was inserted N-terminal to *INO1* to produce the pADH-myc-BioID2-*INO1* and pADH-myc-BioID2(13x)-*INO1* vectors then transformed into *E. coli* and selected for on LB ampicillin (100 µg/mL) agar plates. Vectors were extracted with the NucleoSpin^®^ Plasmid kit (Takara). To produce the un-fused BioID2 expression vector, *INO1* was cleaved out of pADH-myc-BioID2(13x)-*INO1* with XhoI (having cleavage sites at both ends of the gene), ligated with T4 ligase, then transformed into *E. coli*.

In-Fusion cloning of pyPGK18-6xHis-Xpress-MIPS and pyPGK18-6xHis-Xpress-hMIPS was carried out as above where the 6xHis-Xpress-*INO1* and 6xHis-Xpress-*hINO1* fragments were PCR amplified from pRD-*INO1* and pRD-*hINO1*^19^, respectively. In order to construct the pyPGK18-6xHis-MIPS and pyPGK18-6xHis-hMIPS expression vectors, *INO1* and *hINO1* fragments were PCR amplified from pRD-*INO1* and pRD-*hINO1*, respectively, with the 6xHis tag encoded in the upstream primers. All four fragments were inserted into the high-copy, high expression pyPGK18 vector^45^ linearized with EcoRI and PstI (Promega) and transformed into *E. coli*, selecting for positive transformations on LB ampicillin (100 µg/mL) agar plates. All plasmid constructs were confirmed by Sanger sequencing.

Yeast transformations were carried out by high efficiency electroporation as described^46^.

### Cell lysis

Cell culture ODs were taken prior to collecting cell pellets by centrifugation at 3000 × g for 5 min, which were typically stored at −20 °C before lysis. Bead beating with 0.5 mm ZrO_2_ beads (1/2 the lysis buffer volume) was used to break the yeast cell wall in lysis buffers (20 µL/1.0 OD_600_ × mL cells collected) supplemented with ProBlock™ Gold Yeast/Fungi Protease Inhibitor Cocktail (10 µL/mL lysis buffer), 0.5 M EDTA (ProBlock™ Gold kit component) (5 µL/mL lysis buffer), 1 M 1,10-phenanthroline (ProBlock™ Gold kit component) (5 µL/mL lysis buffer), benzonase (0.05 U/µL lysis buffer), and Phosphatase Inhibitor Cocktail 3 (Sigma P0044) (0.2 µL/20 µL lysis buffer), unless stated differently. The metal ion chelators, EDTA and 1,10-phenanthroline, were omitted for nickel ion purifications. A FastPrep-24™ Classic homogenizer was used to vigorously shake samples with beads at 6.5 M/s for 45 s three times, cooling samples on ice for 2 min between runs. Whole cell extracts (WCEs) were clarified by centrifugation at 16,000 × g for 15–20 min at 4 °C. Total protein concentrations were determined using Bradford assay against a standard curve made with known BSA concentrations.

For MIPS protein expression analyses by SDS-PAGE and WB, lysates were collected following a boiling technique for yeast lysis as published^47^, however, we resuspended our cell pellets in 30 µL of 1X Laemmli buffer per 1.0 OD_600_ x mL cells collected and loaded 2 µL of cleared supernatant per lane.

### BioID screen

The BioID screen was carried out as described^29^. Yeast *INO1* deletion mutant cells (*ino1*∆ BY4741) harboring either the pADH-myc-BioID2-*INO1* vector or pADH-myc-BioID2 (unfused BioID2 control) vector were precultured in SCD I+ Leu- selection medium with four replicates per strain. Precultures were washed and used to inoculate 30 mL cultures at a low starting OD_600_ and incubated overnight. Cells expressing fused BioID2-MIPS were grown in SCD I- Leu- selection medium whereas cells expressing unfused BioID2 were grown in SCD I+ Leu- selection medium. When cells reached the mid-log growth phase, cultures were given excess biotin (10 µM) and treated + /− VPA (1 mM) for 1 h. Cultures were then washed twice with cold water to remove excess biotin before proceeding with cell lysis and purification of biotinylated proteins.

### K-CLiP screen

WCEs used for the K-CLiP screen were prepared by cultivating *ino1*∆ + pADH-INO1 in SCD I- Leu- (1 L) to the mid-log growth phase then lysed in HEPES buffer (50 mM HEPES pH 7.5, 150 mM sodium chloride, 10% glycerol, 5 mM imidazole, protease inhibitor cocktail (Sigma P8215) (0.015 µL/µL buffer), benzonase (0.05 U/µL buffer)). These WCEs (1.5 mg total protein) were incubated with kinase buffer (25 mM HEPES at pH 7.5, 50 mM potassium chloride, and 10 mM magnesium chloride) with or without ATP-arylazide (4 mM) at 31 °C for 2 h at 250 rpm with or without UV irradiation (365 nm) in 120 µL total reaction volumes. Enrichment of the crosslinked complexes of 6xHis-Xpress tagged MIPS used nickel magnetic beads (Sigma, 50 µL of bead slurry, washed with HEPES buffer prior to use) and incubation with shaking (1300 rpm) for 30 min at 31 °C. Bound beads were washed twice with wash buffer (500 µL; 50 mM sodium phosphate pH 8.0, 300 mM NaCl, and 10 mM imidazole), and then eluted twice with elution buffer (25 µL each elution; 50 mM sodium phosphate pH 8.0, 300 mM NaCl, and 300 mM imidazole). To analyze the crosslinking efficiency by WB, one third of the K-CLiP samples were separated using a 10% polyacrylamide gel, transferred to a PVDF membrane, and visualized using an Xpress antibody. The remaining two thirds of the K-CLiP samples (1 mg total) were desalted using a 10% polyacrylamide gel by running only 1 cm into the gel. In-gel trypsin digestion was performed as described elsewhere^32^. A Zip tip desalting step was performed after in-gel trypsin digestion with Pierce C18 tips (ThermoFisher, cat. No. 87782) according to manufacturer’s instructions. The samples were dried in a speed vac at −50 °C and stored at −20 °C before proteomic analysis (Wayne State University Proteomics Core).

### LC–MS/MS analysis of K-CLiP generated crosslinked complexes

Dried peptide digests were resuspended in a solution of 5% ACN, 0.1% formic acid, and 0.005% trifluoroacetic acid (20 uL). Samples were separated by ultra-high-pressure reverse phase chromatography using an Acclaim PepMap RSLC column and an Easy nLC 1000 UHPLC system (Thermo). Peptides were analyzed with a Fusion mass spectrometer (Thermo) with a 120,000 resolution orbitrap MS1 scan over the range of m/z 375–1600, followed by ion trap resolution MS2 scans using an m/z 1.6 window and 30% normalized collision energy for HCD. Peak lists were generated with Proteome Discoverer (version 1.4; Thermo), and peptides scored using Mascot (version 2.4; Matrix Science) and Sequest (version 2.1). Label-free quantitation was performed using MaxQuant software.

### SDS-PAGE

Hand cast tris–glycine-SDS gels were used for protein electrophoresis in tris–glycine running buffer (25 mM tris pH 8.5, 190 mM glycine, 0.1% SDS) using the Mini-PROTEAN^®^ Tetra cell electrophoresis system (Bio-Rad). Samples were diluted with 4x Laemmli buffer (0.25 M Tris pH 6.8, 40% (v/v) glycerol, 20% (v/v) 2-mercaptoethanol, 8% sodium dodecyl sulfate, 0.04% bromophenol blue) and water, then heated to 85 °C for 5 min prior to loading on gels.

### Coomassie staining

Total protein staining of gels by Coomassie blue followed the rapid Coomassie staining protocol on Thermo Fisher’s website. Briefly, one mini-gel was submerged in 50 mL staining solution (0.1% Coomassie brilliant blue R, 40% ethanol, 10% acetic acid), loosely covered, heated by microwaving in 15 s increments for a total of 1 min, then incubated with gentle shaking for 15–30 min. The staining solution was discarded, then the gel and container rinsed with deionized water (dH_2_O). The gel was submerged in destain solution (10% ethanol, 7.5% acetic acid), loosely covered, heated by microwaving at 20 s increments for a total of 1 min, then incubated with gently shaking for 30–60 min, reheating once mid-way through. Finally, the destain solution was discarded and the gel incubated with dH_2_O for 5 min prior to imaging.

### Tandem phosphoprotein and total protein staining

Phosphoprotein staining by Pro-Q™ Diamond was performed using an improved staining method^48^ followed by total protein staining with diluted SYPRO™ Ruby^49^ according to the manufacturer’s manual. Imaging of Pro-Q™ Diamond stain used wavelength excitation 515–545 nm and emission 568–617 nm. Imaging of Pro-Q™ Diamond stain used excitation 490–520 nm and emission 568–617 nm.

### Western blotting (WB)

For WB, proteins were transferred from gels to PVDF membranes using the Mini Trans-Blot^®^ system (Bio-Rad) according to the manual. Where applicable, No-Stain™ Protein Labeling Reagent (Thermo Fisher) was used to label total protein according to the user guide. Membranes were blocked with 3–5% BSA prior to antibody probing.

### Phos-tag protein gels and WB modifications

Zn^2+^ Phos-tag™ was cast in 1.0 mm thick bis–tris gels following the Phos-tag™ SDS-PAGE Guidebook and optimized following a published protocol^50^. Importantly, samples were prepared in loading buffer as above, but additionally supplemented with 1 mM zinc chloride. Gel electrophoresis was carried out at 110 V for 1.5 h in 1 L MOPS running buffer pH 7.8 (50 mM MOPS, 50 mM Tris, 0.1% SDS, 10 mM sodium bisulfite). Proteins were transferred from gels to PVDF membranes using a wet-tank method with 1 L transfer buffer (25 mM tris pH 8.5, 190 mM glycine, 10% methanol, 0.1% SDS) under a constant voltage of 30 V for 18 h, followed by an additional 1 h at 100 V. After transfer was complete, membranes were dried to increase protein retention and to reduce background^51^, followed by re-activation with methanol and washing with water twice. Blocking and probing of membranes proceeded using the standard WB protocol above. Notably, use of europium conjugated secondary antibodies and the SpectraMax^®^ i3 ScanLater cartridge (Molecular Devices) was essential for imaging the low-abundance phospho-protein shifts observed in Figs. 1 and 3, as the fluorescent scanner provided a greater dynamic range than chemiluminescence. Mn^2+^ Phos-tag™ was cast in 1.0 mm thick tris–glycine gels following the Phos-tag™ SDS-PAGE Guidebook. This gel chemistry uses the standard tris–glycine-SDS page gel formula (see above) but is supplemented with Phos-tag™ acrylamide and manganese chloride. Samples were prepared in loading buffer as above and supplemented with 1 mM manganese chloride to prevent ion chelation from the gel. Gel electrophoresis was run using a constant voltage of 100 V until the running line exited the gel. Gels were stained following the rapid Coomassie blue protocol.

### Antibodies and imaging

Commercially available primary antibodies used in this study were Xpress Monoclonal Antibody (Invitrogen R910-25; 1:500 dilution). Secondary antibodies anti-mouse and anti-rabbit conjugated to europium (Molecular Devices R8205 and R8204; 1:10,000 dilution each) were imaged using the SpectraMax^®^ i3 ScanLater cartridge. Membranes were dried prior to imaging with the ScanLater cartridge. Secondary antibodies anti-rabbit conjugated to horseradish peroxidase (Invitrogen 31460; 1:10,000 dilution) were imaged using the iBright™ FL1500 Imaging System (Invitrogen). A yeast MIPS-specific custom antibody (anti-yMIPS) was generated by YenZym Antibodies, LLC against a synthesized peptide (amino acids 348–370: SYNHLGNNDGYNLSAPKQFRSKE-Ahx-C-amide) and isolated from rabbit serum by antigen-specific affinity purification. These purified antibodies were diluted in glycerol to 50% and stored at −20 °C; a 1:10,000 dilution of this antibody solution was used in this study.

### Protein purification

Column purification of the four tagged constructs used for in vitro PKC phosphorylation reactions utilized HisPur™ Ni–NTA Resin (2 mL packed resin) according to the manufacturer’s instructions for gravity-flow columns. WCEs were prepared from 500 mL cultures in mid-log growth phase using Ni–NTA equilibrium buffer (PBS pH 7.4 with 10 mM imidazole) as lysis buffer. WCEs were passed through the column twice to enhance binding of tagged proteins to the resin. Resin was washed five times with two-resin bed volumes of Ni–NTA wash buffer (PBS pH 7.4 with 25 mM imidazole) then eluted three times with two resin bed volumes of Ni–NTA elution buffer (PBS pH 7.4 with 250 mM imidazole). Proteins were concentrated and eluant buffer exchanged with MIPS storage buffer (50 mM Tris, 50 mM sodium chloride, pH 7.5) using Amicon^®^ Ultra-4 30 K Centrifugal Filter Devices according to the manufacturer’s user guide. Purified proteins were then aliquoted and stored at −80 °C. Protein concentrations were determined using in-gel protein quantification analysis against a bovine serum albumin (BSA) standard curve. Briefly, concentrated proteins (1.2 µL and 2.4 uL of each purified protein) and a BSA standard curve (0.05–0.8 µg) were loaded and run on a protein gel until running line exited the gel followed by rapid Coomassie blue staining. Proteins were quantified using iBright™ Analysis Software and a linear curve and equation created from the BSA standards, which was then used to calculate the concentrations of the purified proteins.

Batch purification of 6xHis tagged MIPS utilized PureProteome™ Nickel Magnetic Bead (Millipore Sigma LSKMAGH10) following the manufacture’s user guide with some modifications. Suspended beads (50 µL) were washed with equilibration buffer (PBS solution (50 mM sodium phosphate, 300 mM sodium chloride, pH 8) supplemented with 5 mM imidazole and 0.1% Tween 20) prior to incubation with WCEs (lysed in tris buffered saline pH 7.5 with 0.05% Tween 20) for 30 min at 4 °C with gentle vortexing (1300 rpm). Beads were washed four times with washing buffer (PBS solution containing 10 mM imidazole and 0.1% Tween 20) then eluted using elution buffer (PBS solution supplemented with 300 mM imidazole). Eluant buffer was then exchanged with MIPS storage buffer (50 mM Tris, 50 mM sodium chloride, pH 7.5) and concentrated using Amicon^®^ Ultra-0.5 30 K Centrifugal Filter Devices according to the manufacturer’s user guide. 6xHis-MIPS protein concentrations were determined using in-gel protein quantification analysis (as described above) comparing concentrated proteins (5 µL) against a BSA standard curve (0.1–2.0 µg).

Purification of endogenously expressed MIPS was carried out using anti-yMIPS (30 µg) bound to Dynabeads^®^ Protein A (Invitrogen 10001D) (4.5 mg) following the manufacturer’s protocol with some optimization. In order to prevent co-elution of the immunoglobulin heavy chain (approx. 50 kDa) with MIPS (approx. 60 kDa), the antibody was crosslinked to protein A using BS^3^ (bis(sulfosuccinimidyl)suberate) crosslinker (Thermo Fisher 21,580) (1 mM). This concentration was used as higher concentrations reduced the binding capacity of the antibody to its target and were unnecessary for preventing co-elution of the heavy-chain, as previously reported^52^. WCEs lysed in phosphate buffered saline pH 7.4 with 0.1% Tween 20 (PBST) were incubated with the crosslinked antibody for 4 h at 4 °C with gentle vortexing (1200 rpm). Beads were washed five times with PBST and twice with tris buffered saline pH 7.4 with 0.1% Tween 20 (TBST) to prevent carry over of free phosphate, which is incompatible with Phos-tag™ gels. Proteins were eluted with 40 µL 1x loading buffer containing 2 mM MnCl_2_ by heating to 95 °C for 5 min. 20 µL of eluant volumes were used for analysis on Phos-tag™ gels.

Purification of biotin labeled proteins in the BioID screen utilized PureProteome™ Streptavidin Magnetic Beads (100 µL) from WCEs (300 µg total protein) following a modified method for eluting proteins with excess biotin and heat^53^. Briefly, cells were lysed in lysis buffer (50 mM Tris HCl pH 7.4, 150 mM sodium chloride, 0.4% SDS, 1% IGEPAL CA-630, 1 mM EGTA, 1.5 mM MgCl_2_, Protease inhibitor cocktail (Sigma P8215) (0.015 µL/µL buffer), benzonase (0.05 U/µL buffer) by bead beating. WCEs were incubated with streptavidin beads for 30 min at room temperature with gentle vortexing (1300 rpm). Bound streptavidin beads were washed once in lysis buffer (500 µL) for 2 min with gentle vortexing, once in wash buffer (50 mM Tris HCl pH 7.4, 2% SDS), then twice more in lysis buffer. Biotin labeled proteins were eluted twice in 50 µL elution buffer (0.25 mM biotin in mass spectrophotometry grade water).

### LC–MS/MS analysis of purified 6xHis-Xpress-MIPS

Protein Digestion: Samples of 6xHis-Xpress-MIPS enriched by PureProteome™ Nickel Magnetic Beads (see method above) were separated on a 12% SDS-PAGE gel and total proteins stained by SYPRO™ Ruby (see method above). 6xHis-Xpress-MIPS bands were cut out of the gel, reduced with 5 mM dithiothreitol (DTT), then alkylated with iodoacetamide (IAA). Samples were then acidified by adding 12% phosphoric acid (volume added was 1/10 of the total sample volume). Proteins were precipitated by addition of 7 volumes of 90% methanol with 100 mM triethylammonium bicarbonate (TEAB) and precipitates suspended in 50 mM TEAB then digested overnight with 0.4 ug trypsin (Promega).

2D LC–MS/MS: Phosphopeptides were selected from tryptic digests by incubation with TiO_2_ in 2% Trifluroacetic acid (TFA) saturated with glutamic acid in 60% acetonitrile. The TiO_2_ beads were washed three times with 1% TFA in 60% acetonitrile before eluting phosphopeptides with 0.5 M NH_4_OH in 50% acetonitrile. TiO_2_ eluates were neutralized with formic acid, dried under vacuum, and stored at –80 °C until analysis. Both TiO_2_ selected phosphopeptides and non-enriched sample digests were solubilized in 0.1% formic acid then analyzed by LC–MS/MS without further purification. Peptide separation was achieved using reverse phase chromatography (Acclaim PepMap RSLC column, Thermo) under acidic conditions (0.1% formic acid) with an EASY nLC-1000 uHPLC system (Thermo). Peptides were separated over a 70 min gradient and analyzed with an OrbiTrap Fusion mass spectrometer (Thermo). MS1 scans were performed in profile mode in the orbitrap at 240,000 resolution. Data-dependent MS2 acquisitions were accomplished using a 3 s cycle time with abundant ions having charge states 2–7 selected and fragmented using CID at 30% collision energy in the linear ion trap. Maximum injection time was 50 ms and the AGC target was 20,000. Dynamic exclusion was turned on (20 s). A neutral loss trigger of 97.977 was used to select ions for MS3 analysis using ETD fragmentation.

Protein Identification and Quantitation: LC–MS/MS data were analyzed using Proteome Discoverer (Thermo, version 2.4). Peptide identifications were scored using Sequest HT against a reviewed yeast protein database (Uniprot; downloaded on 2021-03-30; 5984 entries) and simultaneously against a matched decoy database to determine the false discovery rate (FDR). The yeast database was modified to include the 6xHis-Xpress epitope tag on the amino terminus of the MIPS protein. Searches allowed up to 2 missed tryptic cleavages and 10 ppm/0.5 Da mass tolerances for parent and fragment ions, respectively. Iodoacetamide derivative of cysteine was specified as a fixed modification. Oxidation of Met and phosphorylation of Ser, Thr and Tyr were specified as variable modifications. A positive identification was achieved with a ≤ 0.1 FDR using the Percolator algorithm. MS2 spectra of phosphopeptides were exported using the Scaffold Viewer proteome software.

### Phosphatase treatment

Phosphatase treatment with lambda phosphatase (Santa Cruz sc-200312A) was performed according to the manufacturer’s instructions. WCEs were treated in 25 uL reaction volumes with 200 units at 30 °C for 1 h. Ovalbumin (Sigma A5503) (0.5 µg) was treated in 7.5 µL reaction volumes with 0–400 units at 30 °C for 1 h. After incubation, reactions were placed on ice and incubated to 85 °C with loading buffer.

### In vitro phosphorylation reactions

The in vitro candidate kinases screen was performed by incubating either 6xHis-Xpress-MIPS (432 ng) or 6xHis-Pah1 (400 ng) with yeast Pho85-Pho80 (200 ng), rat brain protein kinase C (1 nmol/min; Promega), bovine heart protein kinase A (4 pmol/min; Promega), human casein kinase II (pmol/min; New England Biolabs), or human casein kinase I (nmol/min; Promega) for 30 min at 30 °C in a reaction mixture that contained 20 mM Tris–HCl pH 7.5, 10 mM MgCl_2_, and 100 µM [γ-^32^P]ATP (10,000 cpm/pmol; Perkin-Elmer Life Sciences) in a total volume of 20 µL^43,54^. The samples were subjected to SDS-PAGE using a 10% SDS-PAGE gel followed by phosphoimaging and Coomassie blue staining. 6xHis-tagged Pah1 expressed from plasmid pGH313 in *E. coli* strain BL21(DE3)pLysS was purified by affinity chromatography with nickel-nitrilotriacetic acid-agarose^55^, followed by ion exchange chromatography with Q-Sepharose^56^. 6xHis-tagged Pho85-Pho80 protein kinase complex was purified from *E. coli* strain BL21(DE3) expressing from plasmids EB1164 and EB1076 by affinity chromatography with nickel-nitrilotriacetic acid-agarose^57^.

The phosphorylation reactions with CKII and PKC in optimized reaction conditions were performed as follows. Reactions with CKII (NEB P6010) consisted of 20 mM Tris pH 7.5, 10 mM magnesium chloride, 100 µM ATP, purified protein substrate (0.2 µg), and CKII (5 ng)^43,54^, unless otherwise indicated. Reactions with PKCα (abcam ab55672) consisted of 50 mM Tris pH 7.5, 10 mM magnesium chloride, 20 mM calcium chloride, 10 mM 2-mercaptoethanol, 500 µM 1,2-Diacyl-sn-glycero-3-phospho-L-serin, 150 µM 1–2-dioleoyl-sn-glycerol, 100 µM ATP, purified protein substrate (0.2 µg), and PKC (5 ng)^41,54^, unless otherwise indicated. Substrate proteins included purified tagged MIPS (6xHis-Xpress-MIPS, 6xHis-Xpress-hMIPS, 6xHis-MIPS, and 6xHis-hMIPS) or 6xHis-Pah1 (as a substrate control provided by George Carman^58^). Each reaction (20 µL) was incubated at 30 °C for 30 min (unless indicated otherwise). Samples were then placed on ice then incubated for 5 min at 85 °C with loading buffer, which would effectively terminate the reaction. The entire reaction was loaded in a well for SDS-PAGE analysis and gels stained with Pro-Q™ Diamond and SYPRO™ Ruby to quantitate phosphorylation.

### Real-time quantitative PCR (RT-qPCR) analyses

Yeast cultures were collected by pelleting cells by centrifugation at 3000 × g for 5 min. Supernatant was discarded and pellet moved into microcentrifuge tubes by resuspending in 1 mL cold water. Pellet was collected again by centrifugation at 11,000 xg for 1 min and the supernatant discarded. Cell pellets were stored at −80 °C. Lysis was performed by bead beating (see “Cell lysis”) but utilized only the lysis buffer (RLT) supplied and prepared according to the RNeasy^®^ Plus Mini Kit (Qiagen 74,034). RNA was isolated following the kit’s protocol “Purification of Total RNA from Animal Cells”, performing the optional On-column DNases digestion with DNase Set (Qiagen 79,254). RNA concentrations were measured using a NanoDrop 2000c (Thermo Sientific). RNA was reverse transcribed to cDNA with iScript™ Reverse Transcription Supermix (Bio-Rad 1,708,840). RT-qPCR analysis was performed using PowerUp™ SYBR™ Green Master Mix (Applied Biosystems A25741) according to their user guide. The primer pairs targeting *INO1* (forward: GTGAGTTGATGTTAGGTGGCC, reverse: CGGGTGAAATCCTGGTCTTG) and *ACT1* (forward: GATTCTGAGGTTGCTGCTTTG, reverse: TTGACCCATACCGACCATGA) were verified to have a single target and have an efficiency between 90 and 110%. cDNA used in these reactions were diluted 1/10 with nuclease-free water. The RT-qPCR reaction was run using the two-step standard cycling mode (primer Tm ≥ 60 °C) outlined in the PowerUp™ SYBR™ Green Master Mix user guide on a Quant Studio 3 Real Time PCR System.

### Supplementary Information

Supplementary Figures.

Supplementary Table S1.

Supplementary Table S2.

Supplementary Table S3.

## Data Availability

Raw data for LC–MS/MS analyses are supplied in supplementary tables. Additional data will be supplied upon request to the corresponding author, Miriam L. Greenberg.
