# Supplementary material for: Valproate regulates inositol synthesis by reducing expression of myo-inositol-3-phosphate synthase
Source: Sci Rep. 2023 Sep 8;13:14844. doi: 10.1038/s41598-023-41936-2 (PMC10491628; doi:10.1038/s41598-023-41936-2)
Supplement: Supplementary file 1 — Supplementary Figures. [file 41598_2023_41936_MOESM1_ESM.pdf]

# Valproate regulates inositol synthesis by reducing expression of *myo*-inositol-3-phosphate synthase

Kendall C. Case[1], Rachel J. Beltman[2], Mary Kay H. Pflum[2], Miriam L. Greenberg[1*]

[1] Department of Biological Sciences, Wayne State University, Detroit, MI 48202, USA
[2] Department of Chemistry, Wayne State University, Detroit, MI 48202, USA

*Corresponding author
e-mail: mgreenberg@wayne.edu

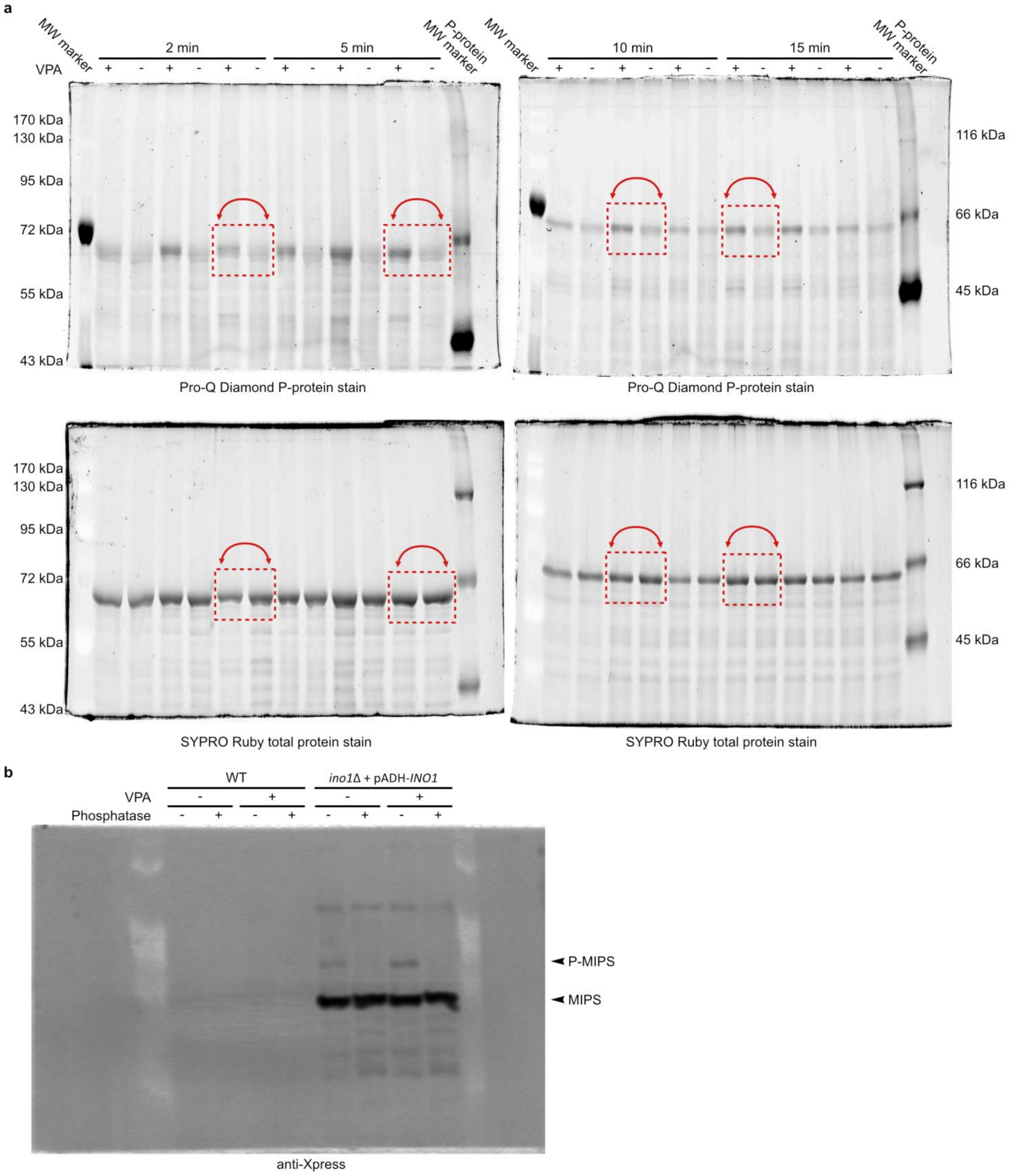


**Supplementary figure S1 Unedited images of figure 1 temporal response (a) and fraction (b) of MIPS phosphorylation following VPA treatment.** (**a**) 6xHis-Xpress-MIPS was purified from yeast treated without or with 1 mM VPA for 2, 5, 10, and 15 min (n=3). Relative phosphorylation of purified proteins was evaluated by running on SDS-PAGE gels followed by tandem staining with Pro-Q™ Diamond phosphoprotein stain then SYPRO™ Ruby total protein stain. Red boxes indicate the cropped regions of the gel which were then flipped so the data could be consistently presented in the -VPA then +VPA order in Fig. 1a. Molecular weight (MW) markers used were Fisher BioReagents™ EZ-Run™ Prestained *Rec* Protein Ladder and PeppermintStick™ Phosphoprotein Molecular Weight Standards. (**b**) WT yeast expressing untagged MIPS or 6xHis-Xpress-MIPS were treated with or without 1 mM VPA for 5 min in the absence of inositol. Cells were lysed and WCEs were treated with or without phosphatase.

**Supplementary figure S1 Unedited images of figure 1 temporal response (a) and fraction (b) of MIPS phosphorylation following VPA treatment continued.** Samples were then analyzed by $Zn^{2+}$ Phos-tag WB, where phosphorylated proteins (P-MIPS) migrate slower than unphosphorylated. WT expresses untagged MIPS and was used to verify the specificity of the antibody. The lower molecular weight bands below the MIPS bands are likely degradation by-products. Shown is the raw membrane image (without contrast adjustment and uncropped) which was used in Fig. 1b. The lanes with white smeared bands contain Fisher BioReagents™ EZ-Run™ Prestained *Rec* Protein Ladder which do not electrophorese well on Phos-tag gels.

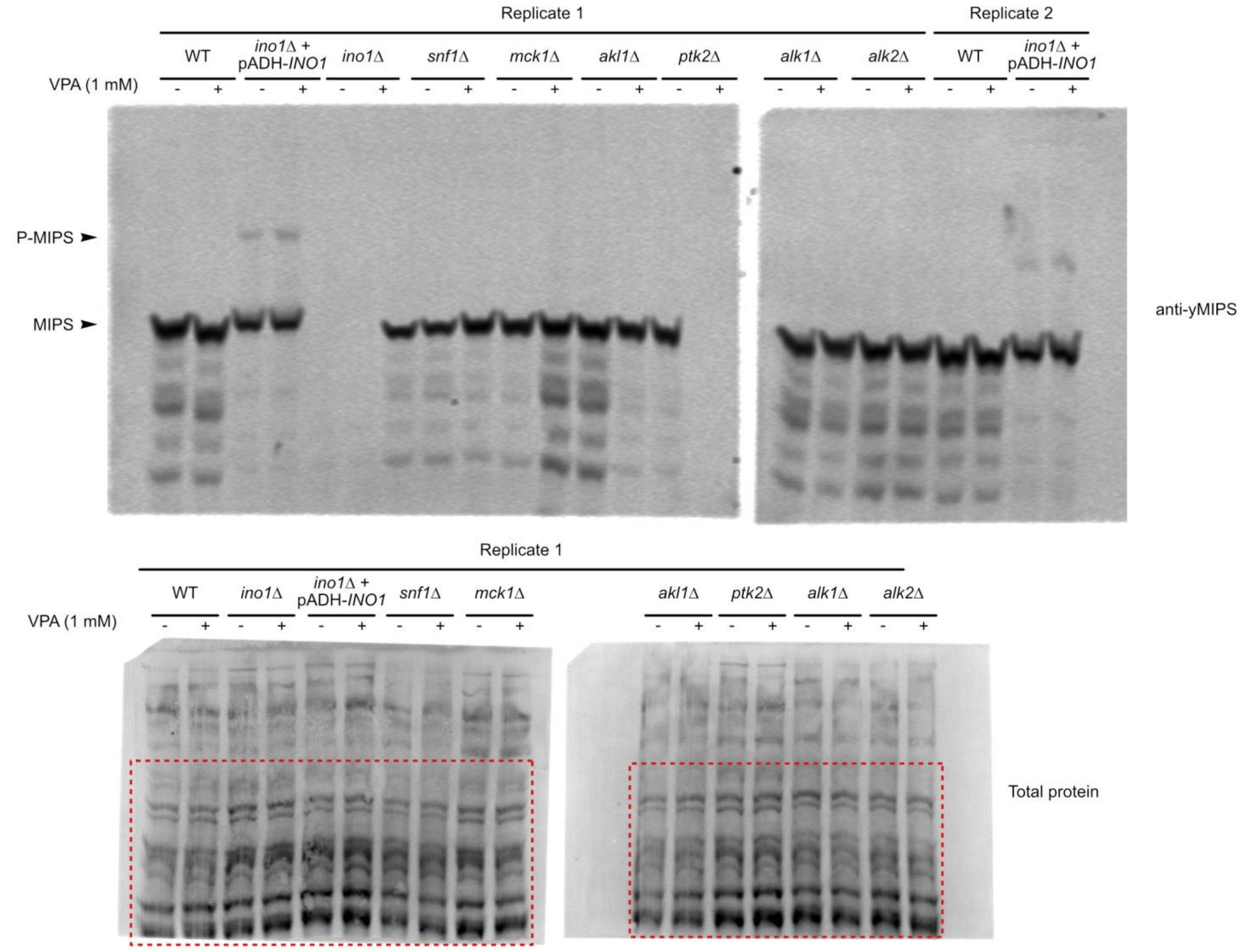


**Supplementary figure S2 Uncropped images of figure 3 only 6xHis-Xpress tagged MIPS is phosphorylated *in vivo*.** Cultures of WT, *ino1*Δ + pADH-INO1, *ino1*Δ, and protein kinase deletion mutants were treated with or without 1 mM VPA for 5 min then WCEs (20 µg total protein) were analyzed by $Zn^{2+}$ Phos-tag WB using an antibody specific for yeast MIPS. MIPS is absent from *ino1*Δ lysates which controls for antibody specificity, confirming that the observed bands are specific to MIPS. A second independent experiment of WT and *ino1*Δ + pADH-INO1 was also assayed to confirm results. To show similar protein quantities between samples, replicate 1 was separately assayed by $Zn^{2+}$ Phos-tag WB and total protein labeled (a separate WB was necessary as we suspected the two imaging techniques may be incompatible). Phosphorylated proteins migrate slower than their unphosphorylated versions. The lower molecular weight bands below the MIPS bands are likely degradation by-products and a few faint non-specific bands. Red boxes indicate the cropped regions of the membranes presented in Fig. 3.

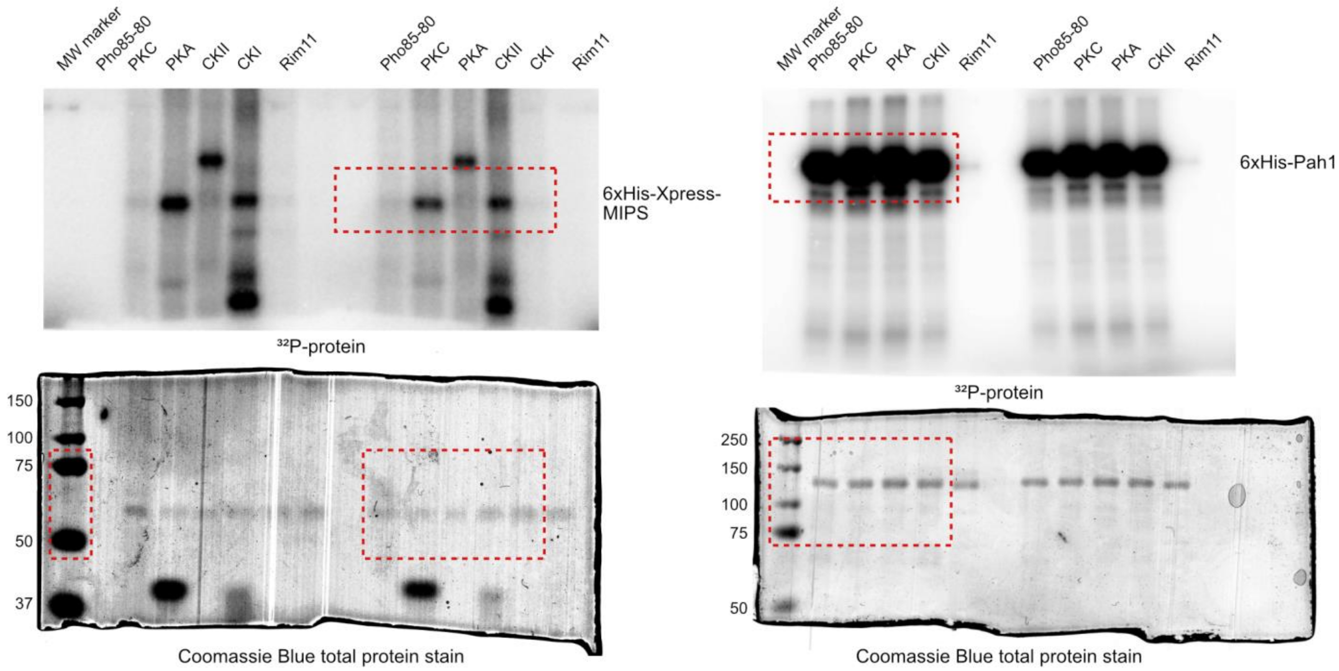


**Supplementary figure S3 Uncropped images of figure 4 *in vitro* phosphorylation of tagged MIPS.** PKC and CKII phosphorylate 6xHis-Xpress-MIPS *in vitro.* Purified 6xHis-Xpress-MIPS was incubated with purified protein kinases in the presence of radioactive [γ-$^{32}$P]ATP. Control reactions to confirm kinase activity utilized 6xHis-Pah1 as the substrate. Reactions were then run on SDS-PAGE gels and phosphorylation imaged by autoradiography (top) and total protein stained by Coomassie blue (bottom)(n=2). 6xHis-Xpress-MIPS has a molecular weight of around 63 kDa. Bands observed at different region of the gel are likely proteins introduced along with the addition of the protein kinases that are phosphorylated by the protein kinases (in the $^{32}$P-protein images) or at a high abundance (in the Coomassie blue stained gels). Red boxes indicate the cropped regions of the gel presented in Fig. 4a. Lanes labeled with Rim11 were excluded from Fig. 4 as this purified protein kinase was later determined to be inactive.

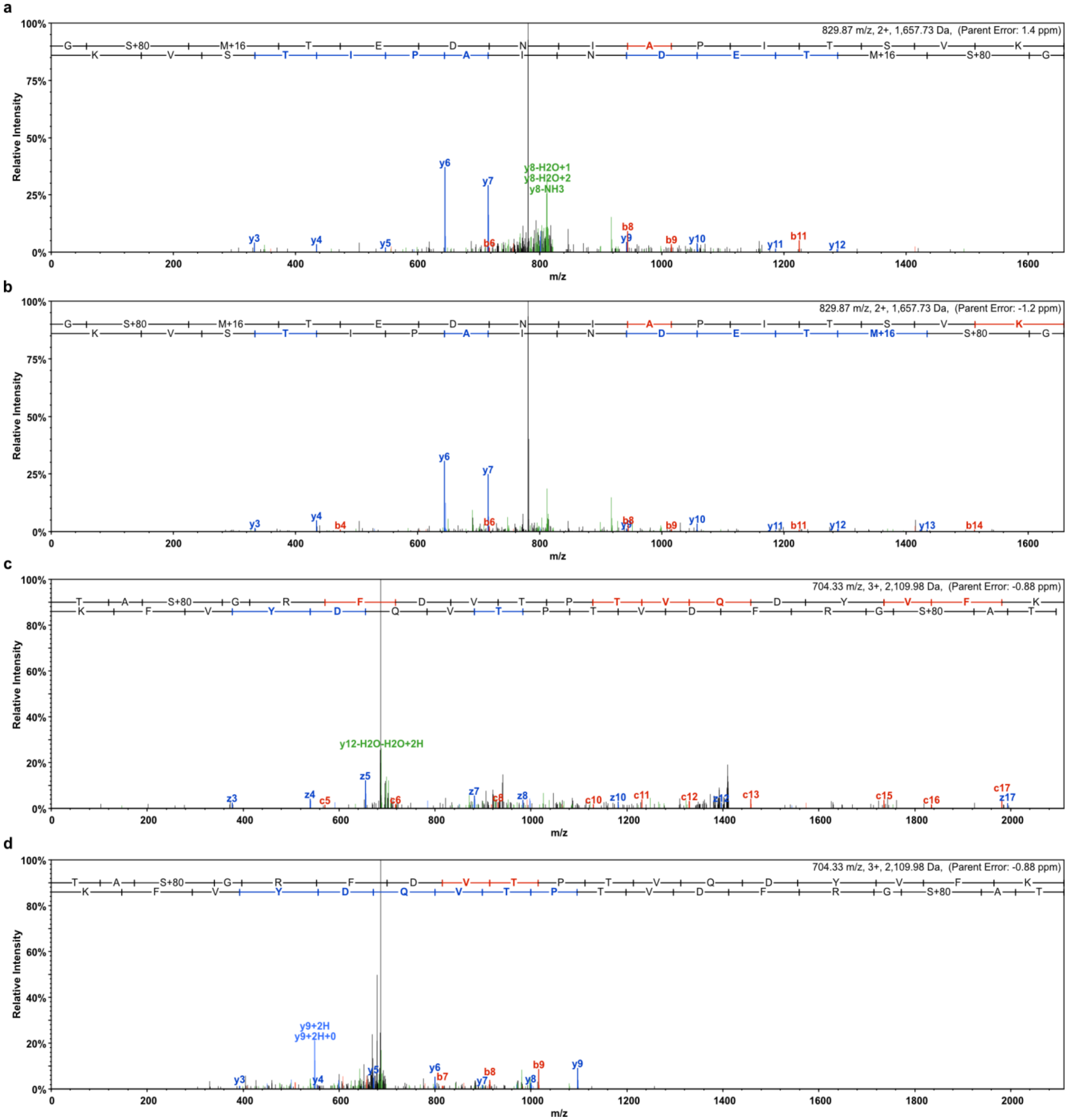


**Supplementary figure S4 MS2 spectra of purified 6xHis-Xpress-MIPS.** Tryptic digests of 6xHis-Xpress-MIPS were analyzed directly (**b**) or after enrichment of phosphopeptides by $TiO_2$ column purification (**a, c-d**). (**a**) Serine phosphorylation of the epitope tag S36 as well as methionine oxidation was identified for the peptide *35-GS*|MTEDNIAPITSVIK-14 in both trypsin-$TiO_2$, with 99% confidence (**a**), and trypsin only samples, with 95% confidence (**b**). The methionine is the initiating amino acid for MIPS protein. Serine phosphorylation of MIPS S43 identified from peptide 41-TASGRFDVTPTVQDYVFK-58 in two fragmentation scans with 70% (**c**) and 44% (**d**) confidence.

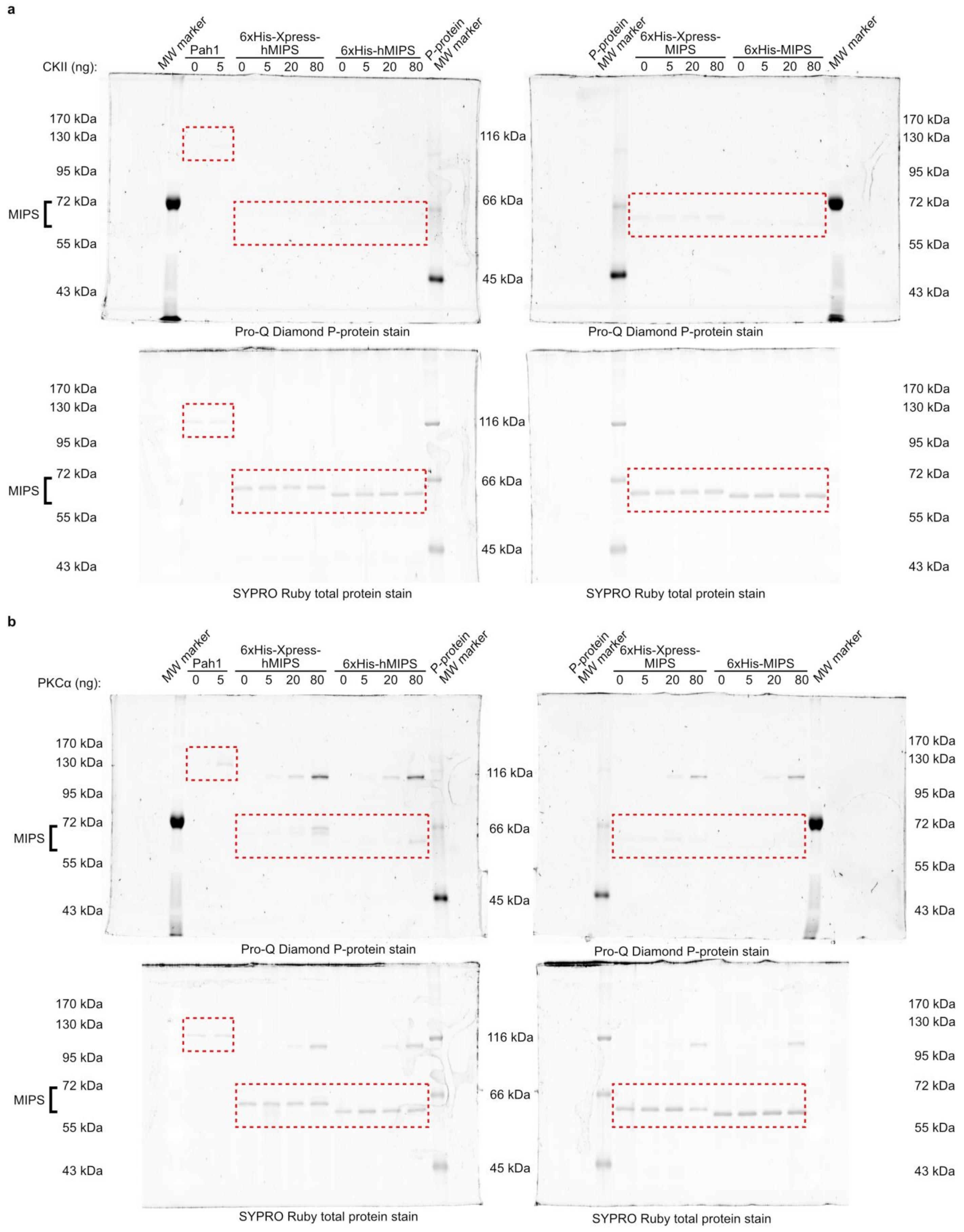


**Supplementary figure S5 Unedited images of figure 5 *in vitro* phosphorylation analyses of different tagged versions of MIPS.** Two differently tagged versions of human and yeast MIPS (hMIPS and MIPS, respectively), one containing the Xpress epitope and

**Supplementary figure S5 Unedited images of figure 5 *in vitro* phosphorylation analyses of different tagged versions of MIPS continued.** amino acids capable of being phosphorylated in yeast (6xHis-Xpress) and the other lacking these (6xHis) (see Fig. 4), were incubated with ATP and increasing amounts of CKII (**a**) and PKCα (**b**). 6xHis-Pah1 was used as a substrate control to confirm kinase activity. Reactions were then assayed by SDS-PAGE and phosphorylated proteins stained with Pro-Q Diamond followed by total protein staining with SYPRO Ruby. Molecular weight (MW) markers used were Fisher BioReagents™ EZ-Run™ Prestained *Rec* Protein Ladder and PeppermintStick™ Phosphoprotein Molecular Weight Standards. The molecular weights of the purified proteins include: 6xHis-Pah1 (120 kDa), 6xHis-Xpress-hMIPS (65.3 kDa), 6xHis-hMIPS (61.9 kDa), 6xHis-Xpress-MIPS (63.8 kDa), 6xHis-MIPS (60.6 kDa), PKCα (103 kDa), and CKII (44 and 26 kDa subunits). The higher molecular weight bands in the PKCα reactions (**b**) are the protein kinases undergoing autophosphorylation. Red boxes indicate the cropped regions of the gel presented in Fig. 5. Image contrast was adjusted for presenting in Fig. 5 so the faint bands can be seen.

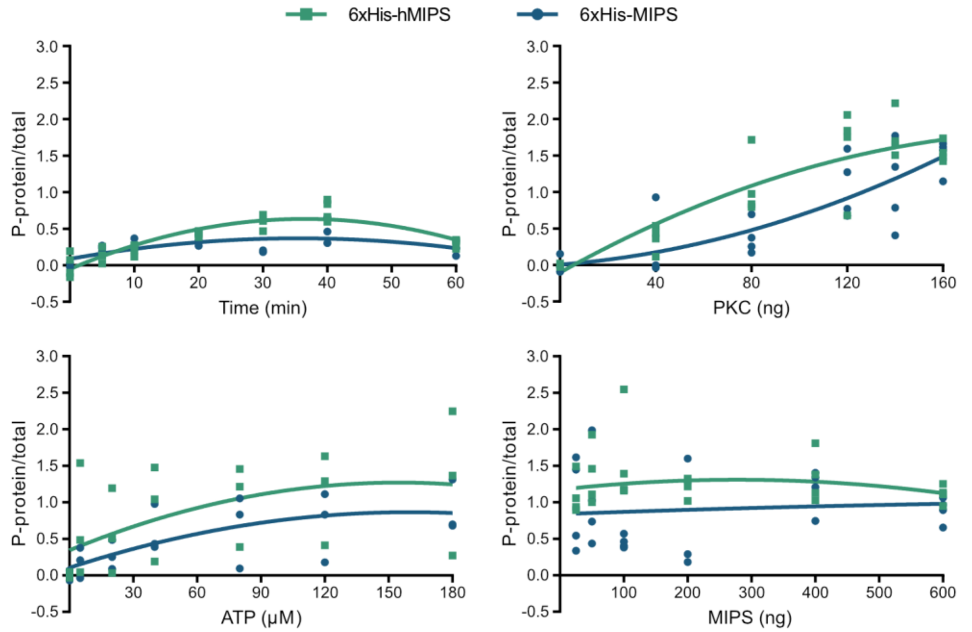


**Supplementary figure S6 Kinetic analyses of MIPS phosphorylation by PKCα.** Short-tagged 6xHis-MIPS substrates (yeast and human) were incubated with PKCα with gradients of time (top-left), kinase (top-right), ATP (bottom-left), and substrate (bottom-right) (n=4). Samples were analyzed by SDS-PAGE and phosphorylated proteins stained by Pro-Q Diamond followed by total protein staining with SYPRO Ruby. Stained gels were quantified, relative phosphorylation calculated (P-protein/total), and a scatter plot generated showing a regression slope calculated with Michaelis-Menten non-linear fit equation. As Pro-Q Diamond phosphoprotein stain has some innate affinity to MIPS, the band densities from the reactions that should not produce phosphorylated MIPS was subtracted as background from the other band density quantitations of that at reaction group.

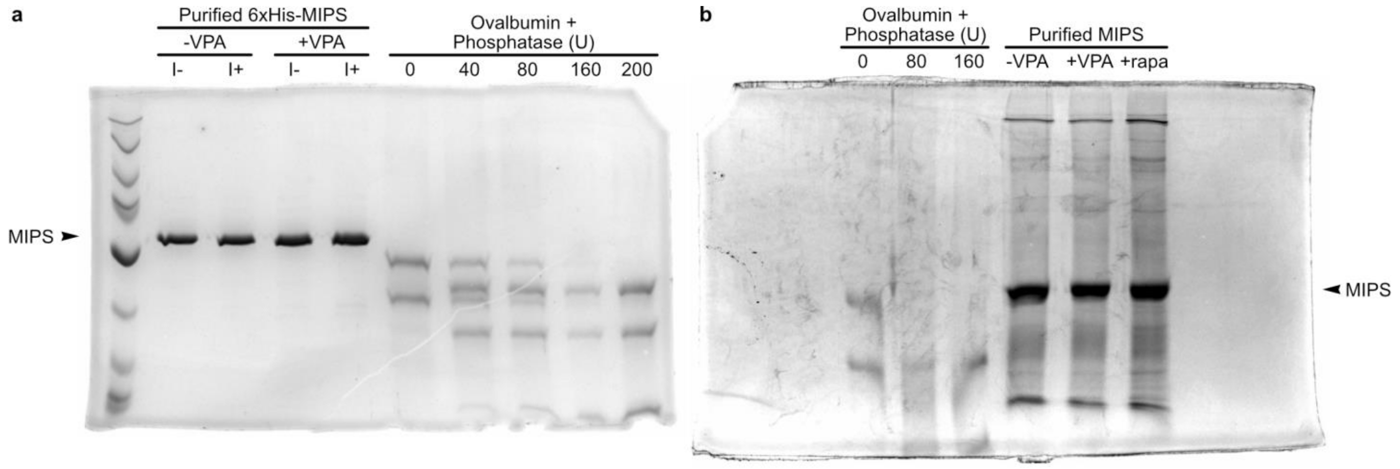


**Supplementary figure S7 Uncropped images of figure 6 *in vivo* phosphorylation of MIPS in response to VPA treatment is undetectable in the absence of the 6xHis-Xpress tag**. (**a**) Yeast cells overexpressing short-tagged 6xHis-MIPS from a vector were treated with or without 1 mM VPA for 1 hr in the presence or absence of inositol. 6xHis-MIPS was isolated from WCEs with Ni-NTA resin by batch purification then concentrated. Phosphorylation of purified proteins (0.5 µg) was determined by $Mn^{2+}$ Phos-tag SDS-PAGE analysis and proteins stained with Coomassie blue. Phosphorylated albumin (0.5 µg) from chicken egg whites (ovalbumin) was treated with increasing amounts of phosphatase and assayed simultaneously to verify Phos-tag gel efficacy. The lane with the molecular weight marker contains Fisher BioReagents™ EZ-Run™ Prestained *Rec* Protein Ladder which does not electrophorese well on Phos-tag gels. (**b**) Cultures of WT yeast were treated with or without 1 mM VPA for 1 hr, then endogenously expressed MIPS immunoprecipitated from WCEs with anti-yMIPS antibodies. Phosphorylation of purified proteins was assayed as before. Phosphorylated proteins migrate slower than their unphosphorylated versions in Phos-tag gels. Rapamycin (400 ng/mL) was also used as a treatment but was excluded from Fig. 6b as it was not discussed in the article.

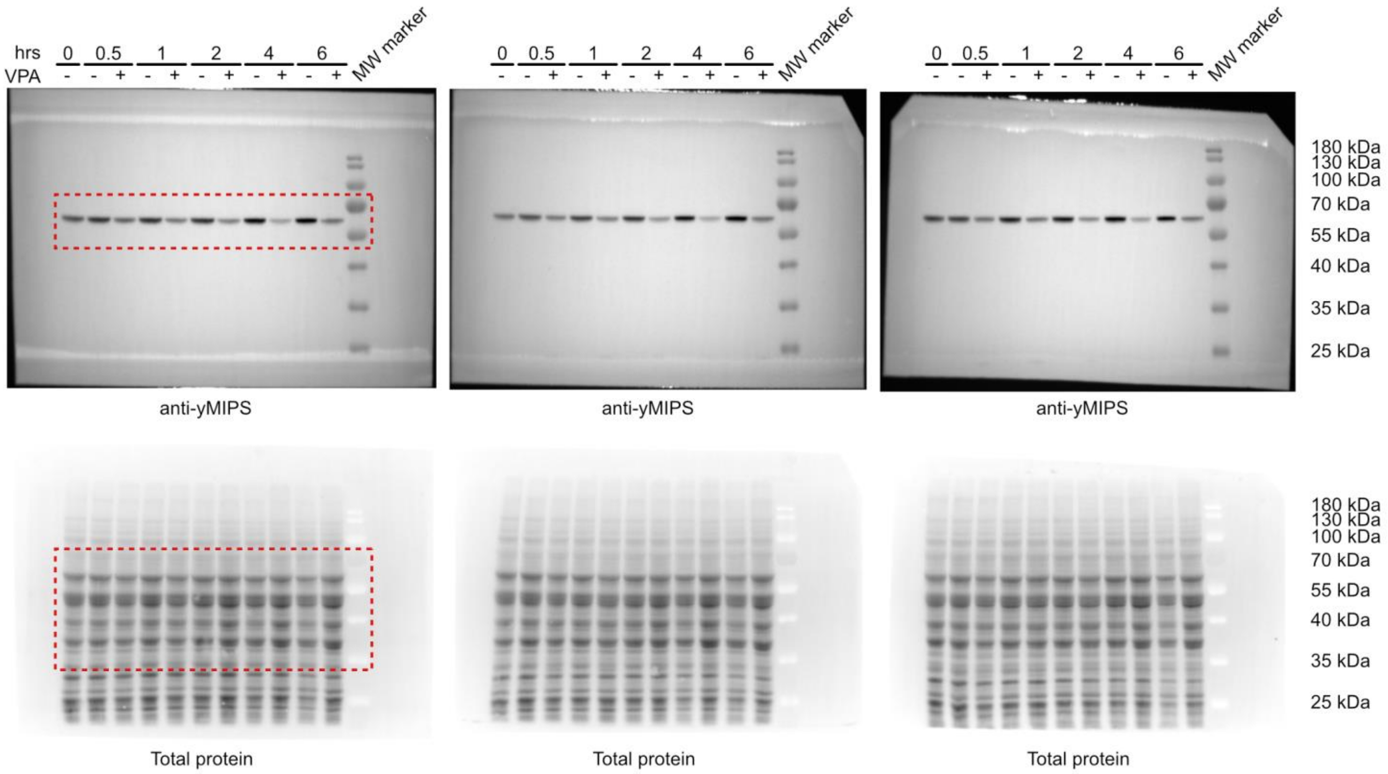


**Supplementary figure S8 Uncropped images of figure 7a MIPS expression is reduced upon VPA treatment.** Yeast cells grown in the absence of inositol to the early-log growth phase were treated with or without 1 mM VPA for up to 6 hrs (n=3). WCEs were analyzed by SDS-PAGE and WB using anti-yMIPS antibodies and a protein labeling reagent to detect total protein. Each biological replicate is loaded on a different membrane. Red boxes indicate the cropped regions of the gel presented in Fig. 7a.
